# Novel stereoselective bufadienolides reveal new insights into the requirements for Na^+^, K^+^-ATPase inhibition by cardiotonic steroids

**DOI:** 10.1038/srep29155

**Published:** 2016-07-05

**Authors:** Hong-Jin Tang, Li-Jun Ruan, Hai-Yan Tian, Guang-Ping Liang, Wen-Cai Ye, Eleri Hughes, Mikael Esmann, Natalya U. Fedosova, Tse-Yu Chung, Jason T. C. Tzen, Ren-Wang Jiang, David A. Middleton

**Affiliations:** 1 College of Pharmacy, Jinan University, Guangzhou city, Guangdong province 510632, P. R. China; 2 Department of Chemistry, University of Lancaster, Lancaster LA1 4YB, UK; 3 Department of Biomedicine, Aarhus University DK-8000, Aarhus, Denmark; 4 Graduate Institute of Biotechnology, National Chung-Hsing University Taichung 40227, Taiwan, China

## Abstract

Cardiotonic steroids (CTS) are clinically important drugs for the treatment of heart failure owing to their potent inhibition of cardiac Na^+^, K^+^-ATPase (NKA). Bufadienolides constitute one of the two major classes of CTS, but little is known about how they interact with NKA. We report a remarkable stereoselectivity of NKA inhibition by native 3β-hydroxy bufalin over the 3α-isomer, yet replacing the 3β-hydroxy group with larger polar groups in the same configuration enhances inhibitory potency. Binding of the two ^13^C-labelled glycosyl diastereomers to NKA were studied by solid-state NMR (SSNMR), which revealed interactions of the glucose group of the 3β- derivative with the inhibitory site, but much weaker interactions of the 3α- derivative with the enzyme. Molecular docking simulations suggest that the polar 3β-groups are closer to the hydrophilic amino acid residues in the entrance of the ligand-binding pocket than those with α-configuration. These first insights into the stereoselective inhibition of NKA by bufadienolides highlight the important role of the hydrophilic moieties at C3 for binding, and may explain why only 3β-hydroxylated bufadienolides are present as a toxic chemical defence in toad venom.

Cardiotonic steroids (CTS) are clinically important drugs for the treatment of heart failure owing to their potent inhibition of cardiac Na^+^, K^+^-ATPase (NKA), the integral membrane protein that maintains ionic gradients in all superior eukaryotic cells^1^. The natural CTS include cardenolides and bufadienolides, known collectively as digitalis, which inhibit NKA by binding with high affinity and selectivity to a “digitalis receptor” pocket extending from the extracellular face of the NKA α-subunit into the transmembrane helical region^2^,^3^. Cardenolides, such as the cardiac glycosides ouabain and digoxin, possess a steroid skeleton bearing a butenolide ring at position C17β and one or more sugar groups at C3^4^. Bufadienolides, which have been isolated from many animals and plants^5^, consist of a steroid skeleton bearing a pentadienolide ring at C17β and can exist as glycosides or aglycones in plants but only aglycones in animals^5^.

Precisely how CTS achieve their remarkably high potency and selectivity for NKA has been a subject of intense investigation for many years^6^. Mutations of the residues in the helix H1–H8 regions all conferred ouabain resistance^7^,^8^. Solid state NMR (SSNMR) studies on several biologically active derivatives of ouabain revealed that the steroid skeleton is considerably more dynamically constrained than the sugar moiety in the NKA binding site^9^. Crystal structures of both high- and low-affinity NKA-ouabain complexes provide snapshots of the inhibitor within its site of action^2^,^3^. The binding pocket of the high-affinity state allows deep ouabain binding with possible long-range interactions between its polarized five-membered lactone ring and Mg^2+^ within a transmembrane coordination site. Recently, the crystal structure of NKA-digoxin complex was reported, which showed a similar conformation to that of ouabain^10^. The crystal structures do not support strong coordination of the sugar moiety of ouabain or digoxin by protein residues close to the extracellular surface, although the possibility that temporary water mediates interactions with the polar residues of the extracellular cavity cannot be excluded.

As compared to the heavily studied cardenolide-NKA binding interactions, the binding mode between bufadienolides and NKA is largely unknown. Bufadienolides ecologically serve as chemical deterrents in many animals and plants as a result of their potent and selective inhibition of NKA^11^. It also showed antitumor effects against various carcinomas^12^ through targeting NKA-associated sigaling pathways^13^. Bufalin (Fig. 1, 1β), a bufadienolide from the venom of various toad species^14^, carries a β–hydroxy group at position C3 instead of a glycosidic linkage. A 3.4 Å crystal structure of the E2P–bufalin-NKA complex refined against anisotropically truncated data suggests that bufalin inserts deeper in the binding site than ouabain and digoxin^10^; however, due to the limited resolution and lack of other comparable complex structures, substantial gaps still remain in our understanding of the chemical requirements for Na^+^, K^+^-ATPase inhibition by bufadienolides.

We recently reported the isolation of a series of new bufadienolides from the venom of *Bufo bufo gargarizans* and found only 3β-hydroxylated bufadienolides^15^,^16^,^17^,^18^. Interestingly, *Bufo bufo gargarizans* synthesizes both 3α- and 3β-hydroxylated bufalin, as both isomers were firstly found to occur in the heart in a 2:3 ratio and blood in a 1:2 ratio (Fig. 2 and supporting information, SI, Figures S1-1 and 2), yet only the 3β-isomer is secreted in venom (SI, Figure S1–3). This observation led us to investigate whether the configuration of the 3-hydroxy group of bufalin or the nature of substituents influences the inhibitory activity against purified NKA.

## Results

### Synthesis of four pairs of diastereomers of bufalin (1α–6β)

**Figure 1:**
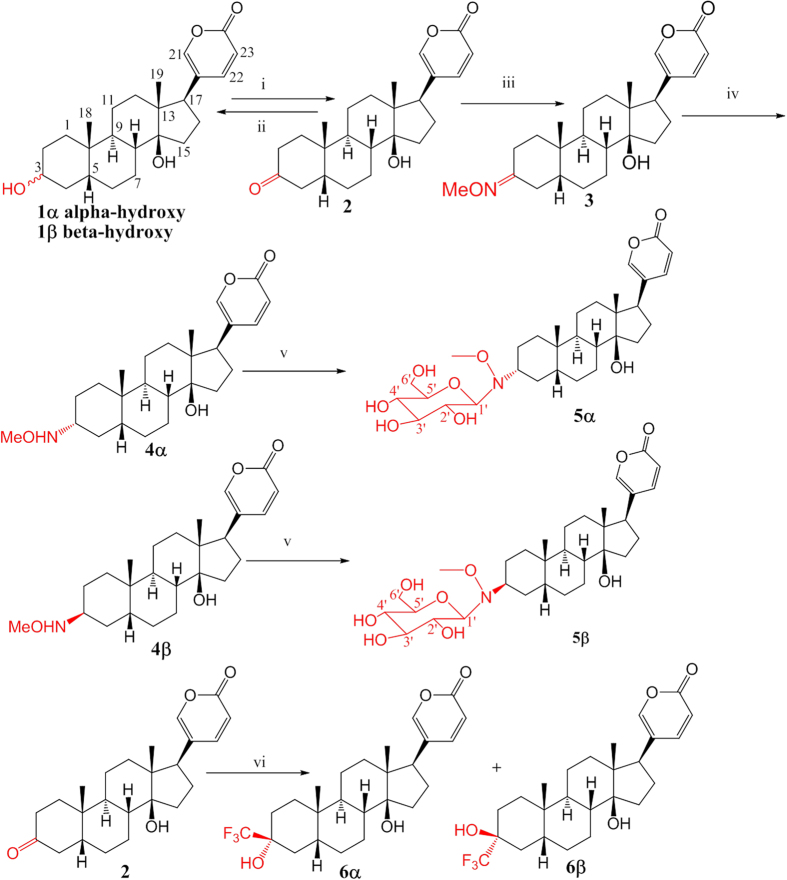
Structures and synthetic scheme for the four pairs of diastereomers of bufadienolides at C3. Reaction conditions: (i) PCC, rt, 4 h; (ii) NaBH_4_ in THF; (iii) MeONH_2_.HCl in pyridine and methanol; (iv) *t*-BuNH_2_BH_3_.HCl, 0 ^°^C, 3 h; (v) ^13^C-glucose, DMF/AcOH, 40 ^°^C, 48 h and (vi) *n*-tetrabutylammonium fluoride and Trimethyl(trifluorometylhyl)silane in THF, followed by treatment of CsF in methanol**. 5α** and **5β** were ^13^C-labelled at the numbered positions for solid-state NMR analysis.

We designed and synthesized four pairs of isomers of bufalin through inversion of the configuration at C3 (**1α** and **1β**, Fig. 1) and introduction of hydrophilic (methoxyamine derivatives **4α** and **4β** and glycoside derivatives **5α** and **5β**) or hydrophobic groups^19^ (trifluoromethylated derivatives **6α** and **6β**) at C3. These probes are suitable for us to investigate whether the configurations and nature of the substituents at C3 influence the inhibitory activity.

The starting material bufalin (**1β**) was purified from the powdered venom (1.0 kg) of *Bufo bufo Gargarizans* with a yield 0.465%. Derivatives **1α**, **4α, 4β**, **5α** and **5β** were synthesized from bufalin (**1β**, Fig. 1) using a neoglycosidation approach (supporting information, SI)^20^,^21^. 3*R*-bufalin (**1α**) was synthesized by PCC oxidation followed by a reduction with NaBH_4_. In the ^1^H-NMR spectrum of **1α**, the oxygenated methine at C-3 resonates at δ 3.65 with a multiplet pattern, which indicates that the hydroxyl group should be α-oriented because the β-oriented proton H-3, adopting the axial position, is split by two axial protons (H-2α and H-4α) and two equatorial proton (H-2β and H-4β) resulting large *aa* and small *ae* couplings. The configuration of H-3 in **1α** was further confirmed by NOESY, which showed that H-3 was correlated to H1β, H-5 and the β-oriented H_3_-18 (the methyl at C-10). In contrast, In the ^1^H-NMR spectrum of **1β**, the oxygenated methine at C-3 resonates at δ 4.13 with a broad singlet pattern, which indicates that the hydroxyl group should be β-oriented because the α-oriented proton H-3, adopting the equatorial position, is split by adjacent protons resulting either *ea* or *ee* small couplings and appears as a broad singlet.

The aglycone diastereomers **4α** and **4β** were obtained in a 2:1 ratio by reaction of bufalone (**2**) with methoxyamine followed by reduction with *tert*-butylamine·borane complex. Similar to **1α** and **1β**, in the ^1^H-NMR spectrum of **4α**, the oxygenated methine at C-3 resonates at δ 2.91 with a multiplet pattern, which indicates that the proton should be β-oriented and adopt the axial position, which is split by two axial protons (H-2α and H-4α) and two equatorial proton (H-2β and H-4β) resulting large *aa* and small *ae* couplings. In contrast, the ^1^H-NMR spectrum of **4β** showed that the oxygenated methine at C-3 resonates at δ 3.24 with a broad singlet pattern, which indicates that the proton should be α-oriented, and adopt the equatorial position.

**Figure 3:**
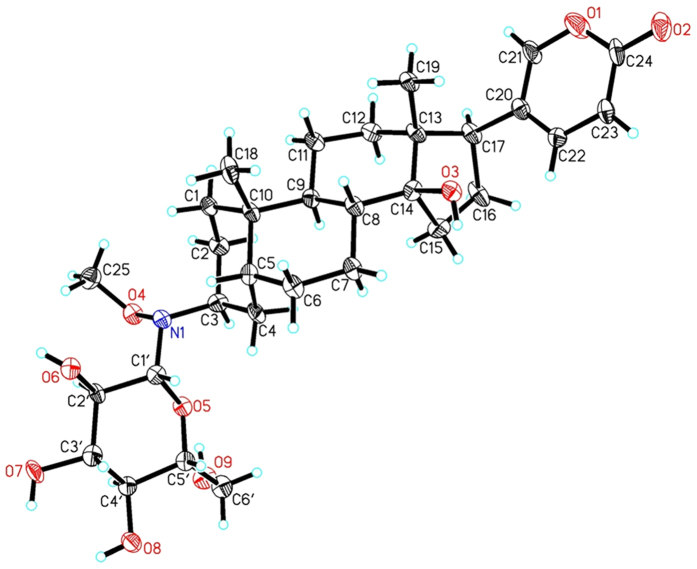
X-ray structure of 5β with atom labelling scheme.

Compounds **5α** and **5β** were ^13^C-labelled glycosides as shown to permit solid-state NMR analysis of the NKA-inhibitor complexes. These two compounds were synthesized by reaction of aglycone **4α** and **4β**, respectively, with ^13^C-labeled D-glucose (U-^13^C6, 99%) in mixed solvents DMF/AcOH (3:1), and the final products were purified by preparative HPLC. The ESI-MS spectra of both glycoside diastereomers **5α** and **5β** showed pseudomolecular ions at m/z 584.5 [M+H]^+^, 606.4 [M+Na]^+^ and 1189.4 [2M+Na]^+^ corresponding to a molecular formula ^12^C_25_^13^C_6_H_47_NO_9_. Similar to **1α**/**1β** and **4α**/**4β**, the configuration at C-3 of **5α**/**5β** can be assigned by chemical shifts and spectra splitting [**5α**: δ3.66 (m, 1H); **5β**: δ3.88 (brs, 1H)]. The configuration of **5α** could be further confirmed by NOESY spectrum, which showed that H-3β is correlated to H_3_-18, and the configuration of **5β** was confirmed by single-crystal X-ray analysis (Fig. 3) which showed two independent molecules in the asymmetric unit (SI, Figure S7-4).

Synthesis of 3-trifluoromethyl derivatives **6α** and **6β** was achieved by reaction of bufalone (**2**, 0.2 mmol) with Trimethyl(trifluorometylhyl)silane (1.0 mmol) catalyzed by *n*-tetrabutylammonium fluoride followed by a deprotection with CsF (5eq.) in methanol. For compound **6α**, the CF_3_ group occupies the axial position and thus have higher energy (73.08 kcal/mol) than that of **6β** (70.45 kcal/mol). Thus the yield of compound **6α** is lower than that of **6β**. Furthermore, occupying the axial position the CF_3_ group of **6α** possesses higher electron density than **6β**. Accordingly, the fluorine of **6α** has larger negative chemical shift (δ −2.98) as compared with **6β** (δ −77.08). In addition, the NOESY spectrum collected in DMSO-*d*_6_ of **6β** showed correlation between 3-OH and H_3_-18, confirming that 3-OH is β-oriented. In contrast, the corresponding correlation was absent in the NOESY spectrum of **6α**, suggesting that 3-OH in **6α** is α-oriented.

All the final products were purified by preparative HPLC. The final purities for all products were over 98%. Details of the spectral data of four pairs of isomers and all other inhibitor syntheses are given in the supporting information (SI and Figures S2-1 to S9-4).

### Inhibitory activities of the four pairs of diastereomers of bufalin on NKA

**Figure 4:**
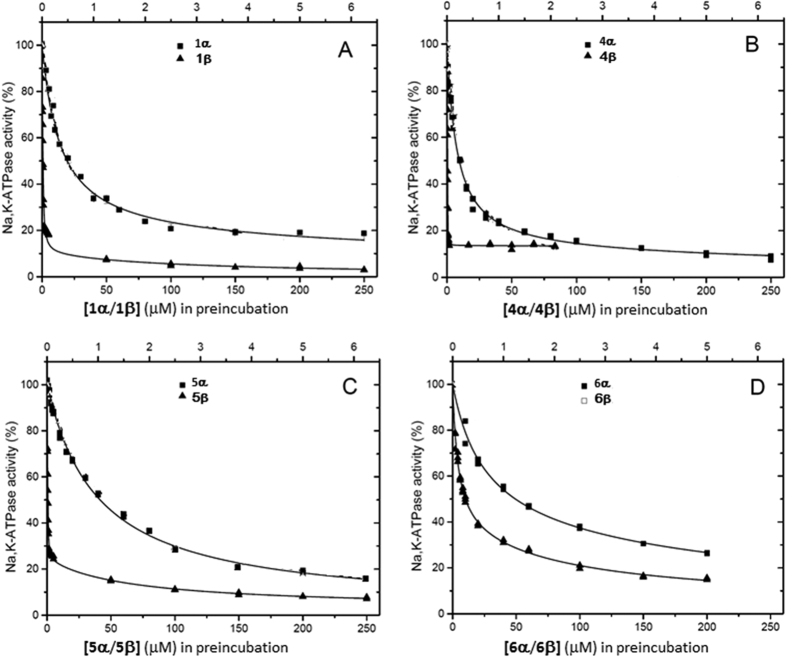
The inhibitory potencies of bufadienolides against renal NKA at 37 °C. (**A**) Dose-response curves showing the ATPase activity remaining at the specified concentrations of 3α-bufalin (**1α**) and 3β-bufalin (**1β**); (**B**) Curves for **4α** and **4β**. (**C**) Curves for **5α** and **5β; (D)** curves for **6α** and **6β**. Symbols ◾ used for α-isomers and ▴ for β-isomers. The solid lines represent hyperbolic fits to the inactivation measured after preincubation of the inhibitors with NKA for 2 hours at 37 °C in the presence of Mg^2+^ and Pi. Expanded views of the low concentration regions of the curves are given in SI, Figure S10.

The inhibitory potencies of bufalin (**1β**, a natural bufadienolide) and its 3*R* isomer **1α** were determined from the residual NKA hydrolytic activities after incubation with varying drug concentrations using our reported method^9^,^10^ (Fig. 4A). The residual rates of ATP hydrolysis were plotted vs. different drug concentrations and analysed by fitting hyperbolic functions corresponding to a dominant high-affinity component and (where appropriate) a second smaller low-affinity component, and the resulting kinetic parameters were shown in Table 1. See supporting information section 12 for experimental methods and details of the kinetic analysis. Bufalin **1β** showed potent inhibitory activity with a K_Diss,high_ value of 0.25 ± 0.02 μM for the high affinity component. Remarkably, inversion of the hydroxyl group (**1α**) resulted in a considerable loss of inhibitory potency, with the ratio of K_Diss,high_ values being 55 for **1α**:**1β** (Fig. 4A and Table 1).

We explored whether an *S* configuration at C3 actively enhances function (e.g., by allowing the hydroxyl group of **1β** to hydrogen bond within the binding site) or whether an *R* configuration actively diminishes function (e.g., by causing steric clashes of **1α** with binding site residues). The hydroxy groups of **1α** and **1β** were replaced with larger methoxyamine groups alone (**4α** and **4β**) or with glycoside groups (**5α** and **5β**) using a neoglycosidation approach (Fig. 1)^19^,^20^. CTS of the cardenolide class, such as ouabain and digoxin, carry one or more glycosidic groups at C3 that serve to increase aqueous solubility and generally enhance potency, although their activity also depends on the chemical structure of the glycosidic substituent^22^,^23^. **4β** and **5β** both showed somewhat higher inhibitory activity than the natural product bufalin (Fig. 4B,C), with K_Diss_ of less than 0.10 μM for the major high-affinity inhibitory component (Table 1). By contrast **4α** and **5α** were considerably less active than bufalin and their 3*S* counterparts, with the ratio of K_Diss,high_ values being 145 for **4α**:**4β** and 461 for **5α**:**5β** (Table 1). The rank order of K_Diss,high_ values **1α**:**1β **< **4α**:**4β **< **5α**:**5β** reflects the varied selectivity of the three pair of isomers. Hence the stereoselective trend appears to arise from both enhanced stabilizing interactions of the enzyme with the increasingly large 3*S* polar groups and destabilizing steric clashes with the increasingly large polar groups in the 3*R* configuration. It is noteworthy that as observed for bufalin, fitting of the inhibition curves indicates the presence of a minor low affinity component. Although there is some apparent variability in the proportion of this component and in the value of K_Diss,low_ for the different inhibitors, the errors are too large to allow meaningful comparisons. Interestingly, a further pair of derivatives, **6α** and **6β**, in which the hydrogen at the C3 position of **1α** and **1β** was replaced with CF_3_, are both considerably poorer inhibitors than bufalin (Fig. 4D), and the stereoselective effect is rather modest (**6α**:**6β** = 6.4). Hence the introduction of the hydrophobic group^19^ at C3 is detrimental to activity in both the 3*S* and 3*R* forms.

### Insight into the interactions of between ^13^C-labelled glycosyl diastereomers and NKA using solid-state NMR

**Figure 5:**
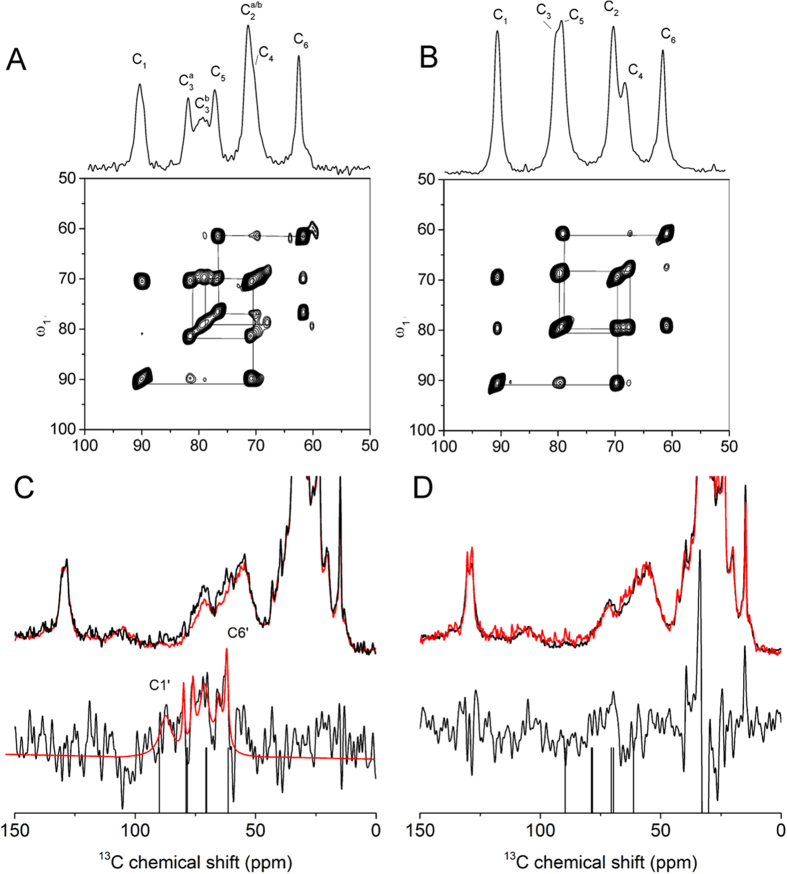
^13^C CP-MAS SSNMR spectra of NKA membranes containing glycosyl-bufalin derivatives 5α and 5β. Panel A,B are DARR spectra of solid **5β** and **5α**, respectively. Panel C shows spectra for **5β** with NKA and panel D shows spectra for **5α** with NKA. The red spectra at the top of each panel are for enzyme without inhibitor and the black spectra are for enzyme with inhibitor. Difference spectra (5 × vertical expansion) are shown at the bottom of each panel and the difference spectrum for **5β** has been fitted with 6 Lorentzian lines centred at the estimated chemical shifts for C1’-C6’. The drop lines indicate the resonance positions in the proton-decoupled ^13^C spectra of the compounds in aqueous solution.

We next considered whether the enhanced activity of **5β** arises from the additional hydrogen-bonding capacity of the glucose moiety. Crystal structures of NKA complexed with cardenolide glycosides^2^,^3^,^10^ and SSNMR measurements of ouabain derivatives^9^ suggest only a loose association of the sugar moiety with residues in the binding site^10^. Here we used ^13^C cross-polarization magic-angle spinning (CP-MAS) SSNMR to report on the interactions of the glycosidic groups of **5α** and **5β** with the binding site. Figure 5A,B shows dipolar-assisted rotational resonance spectra of solid **5β** and **5α** confirming the ^13^C labelling pattern. In Fig. 5 panels C and D are shown ^13^C CP-MAS SSNMR spectra of NKA (13 nmol) in the absence of inhibitor (red) and with **5β** or **5α** (each 16 nmol) (black), respectively. From Table 1 it is estimated that there is 99.5% saturation of the binding site by **5β** and about 75% saturation by **5α**. For the complex with **5β** (Fig. 5C), signals from the inhibitor can clearly be observed around 60–90 ppm; these signals are absent when NKA is preincubated with ouabain before adding **5β** (SI, Figure S11-1), which confirms that ouabain blocks the binding site for **5β**. Signals from unbound inhibitor are not observed under the measurement conditions (see, e.g., ref. 24). The chemical shifts (measured with assistance from a DARR spectrum; SI, Figure S11-2) are considerably different from the solution state values, although the spectrum could only be assigned tentatively (Table 2). The signals indicate that the glucose moiety of the inhibitor is dynamically restrained as a result of interacting with the enzyme and the distinct chemical shifts are consistent with the glucose moiety undergoing polar/hydrogen bonding interactions with residues within the binding site. By contrast, signals from **5α** are not observed in the presence of NKA (Fig. 5D), indicating that the glucose moiety of the 3*R* isomer is not motionally restrained by the enzyme.

### Understanding the stereoselective effects of bufadienolides on NKA using computational molecular docking analysis

**Figure 6:**
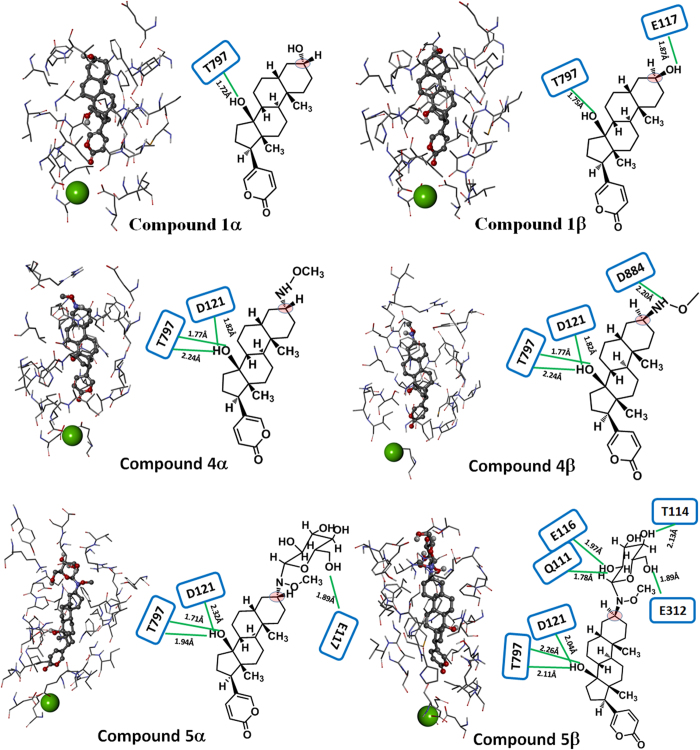
Detailed molecular interactions between the binding pocket of NKA and bufadienolides. For each compound, the amino acids of NKA close to the ligand compounds (ball-and-stick structure) are shown in stick structure. Mg^2+^ close to the ligand is shown in CPK (green ball). Amino acid residues of NKA involved in formation of hydrogen bonds are shown in blue squares. Distances of hydrogen bonds (green lines) between ligands and NKA (from donor hydrogen to receptor) are indicated. The pink circle was used to highlight the configuration difference in C-3 position.

The origin of the stereoselective effect of **1**, **4** and **5** was explored with computational molecular docking analysis, using the crystal structure of the NKA-ouabain complex to initially position the inhibitors (Fig. 6). In the high-affinity complex^3^, the cardenolide glycoside ouabain inserts into a binding pocket between transmembrane helices and is stabilized by polar interactions on one face and nonpolar interactions on the other face, with possible long-range interactions between its polarized five-membered lactone ring and Mg^2+^ within a transmembrane coordination site. The structure of NKA complexed with digoxin showed a similar binding location to that of ouabain^10^, whereas a lower-resolution structure of the bufalin-NKA complex suggests that the aglycone bufalin inserts deeper into the site^10^. Here, docking models of **1α** and **1β** indicated that an H-bond forms between the hydroxyl group at C14 and T797 of NKA. In contrast, three H-bonds were formed between the hydroxyl group at C14 of **4α, 4β**, **5α** and **5β** and D121/T797 of NKA. The inversion at C3 is predicted not to compromise the hydrogen bonding interactions deeper within the binding site. The major differences in the interactions of the three pairs of isomers within the binding pocket of NKA occurred in the regions of the hydrophilic moieties at C3. The hydroxyl group at C3 of compound **1α** has no clear hydrogen-bonding partner, whereas one H-bond was formed between E117 of NKA and the hydroxyl group at C3 of compound **1β**. No H-bond was observed for the α-configured methoxyamine at C3 of compound **4α**, whereas one H-bond was formed between the β-configured methoxyamine at C3 of compound **4β** and D884 of NKA. Only one H-bond was formed between α-configured glycoside at C3 of compound **5α** and E117 of NKA; while four H-bonds were formed between β-configured glycoside at C3 of compound **5β** and Q111/T114/E116/E312 of NKA. Hence the hydrophilic groups at C3 with β-configuration (**1β, 4β** and **5β**) appear to be closer to the hydrophilic amino acid residues in the entrance of the ligand-binding pocket than those with α- configuration (**1α, 4α** and **5α**) and this may account for the higher affinities of the β-isomers.

## Discussion

In this study, we found that the configuration and chemical nature of the C3-substituent of bufadienolides are critical for inhibition of NKA. Remarkable selectivity is observed, in that replacement of the 3β-hydroxyl of bufalin with larger polar groups in the same configuration results in enhanced inhibitory potency, yet activity is diminished simply by inversion of the 3β-hydroxyl to 3α or by adding a small hydrophobic group in either configuration. Interestingly, 3β-substituted neoglycosyl derivatives of the cardenolide class show enhanced antitumour activity compared with their 3α isomers, yet the 3β-isomers showed considerably weaker inhibition of NKA^21^ than we report here for the bufadienolides, which might be due to different bioassay methods. Our observations suggest that 3β-hydroxylated bufadienolides are secreted preferentially in venom because they have stronger inhibitory potency against NKA and thus act as a more effective toxic chemical defence against predators^25^,^26^.

## Materials and Methods

### Ethics statement

All experimental protocols were approved by the Jinan University Institutional Review Board. Specifically, the animal experiments were conducted in accordance with the Guide for Care and Use of the Laboratory Animals published by the Jinan University (publication SYXK2012-0117).

### UPLC detection of bufadienolides in toad heart and blood

Seven toads (*Bufo bufo Gargarizans*) were anesthetized, and blood was drawn (total 3.5 ml) and the hearts were removed and homogenized (3.8 g). The methanol extract of the heart homogenate and blood sample were partitioned by dichloromethane against water, respectively, and were further purified by a solid phase extraction, which was eluted firstly with water to remove the high polar constituent and then methanol (2 ml) was used elute the organic compounds. The methanol elute was filtered through a 0.22 μm PTFE syringe filter, and an aliquot of the filtrate (10 μL) was injected in the UPLC instrument for analysis. The two peaks at *R*_t_ = 10.62 min and *R*_t_ = 10.85 min were identified by ESI-MS and confirmed by comparison with the standards (Figures S1-1 and S1-2). The chemical profiles of the heart homogenate and blood were compared with that of the total bufadienolides (0.1mg/mL, injection 5.0 μL) of the venom of *Bufo bufo gargarizans* by UPLC analysis (SI, Figure S1-3).

### Synthesis of four pairs of diastereomers of bufalin (1α–6β)

Details of the procedures for the synthesis of four pairs of diastereomers of bufalin, the spectral data and configuration assignments are given in the supporting information (SI and Figures S2-1 to S9-4).

### Na^+^, K^+^-ATPase inhibition assay

Na^+^, K^+^-ATPase from pig kidney microsomal membranes was purified by differential centrifugation^27^. The specific activity of the enzyme preparation was approximately 30 μmol ATP hydrolysed/mg protein per min at 37 °C (see SI section 11). The inhibitory effects of bufadienolides on NKA were determined essentially as previously reported^9^,^10^. A detailed kinetic analysis is given in the SI section 12.

### Solid-state NMR experiments

One dimensional proton-decoupled ^13^C CP-MAS NMR experiments were performed at −25 °C using a Bruker Avance 400 MHz spectrometer at a magnetic field of 9.3 T equipped with a 4 mm HXY probe and (for 2D measurements on membranes) on a Bruker Avance III 700 MHz instrument with a 3.2 mm HXY probe operating in double-resonance mode. Samples were spun at a MAS rate of 5 kHz in a 4 mm zirconium rotor. Hartmann-Hahn cross-polarization was achieved with a 1.6-ms contact time and 83 kHz proton decoupling with SPINAL-64 was applied during signal acquisition. Each spectrum was the result of accumulating 100,000–200,000 transients with block averaging. Two-dimensional DARR spectra of solid inhibitors were recorded at room temperature with a 2-ms contact time at a field of 63 kHz, 83 kHz SPINAL-64 decoupling, 5-ms recycle delay and a 8-kHz proton field matched to the spinning frequency during a 10-ms mixing time. The time domain matrix was the result of 128 t_1_ increments, each averaged over 64 transients. Phase-sensitivity was achieved using the States-TPPI method. The DARR spectrum of the 5b-NKA complex was acquired at −25 °C with 14 kHz spinning, 20-ms mixing time and 64 t_1_ increments averaged over 4096 transients and a 1.6 –ms recycle delay.

### Molecular modeling and docking

The crystal structure of pig kidney NKA-ouabain complex with Mg^2+^ (PDB code 4HYT) was downloaded from Protein Data Bank and used for the molecular modeling and docking^3^. Ouabain in this complex structure was removed first, and the modified NKA after hydrogen saturation was applied with CHARMm force field using the Discover Studio 2.1 package (http://accelrys.com/products/discovery-studio/). The 2D structures of compounds **5α** and **5β** used in this study were constructed by using the ChemDraw program, and their corresponding 3D structures were converted by the Chem3D program (http://www.cambridgesoft.com/). The binding site for the ligand compounds in the Na^+^, K^+^-ATPase α-subunit was defined as ouabain binding site. In the docking simulation of ligand compounds, the binding domain was defined as the region of the sphere with a 12.5 Å radius from the center of the binding pocket. Docking of ligand compounds was performed *in silico* by employing the LibDock module in the Discover Studio 2.1 package, and further minimized by smart minimize algorithm with CHARMm force field in the Discover Studio 2.1 package^28^. In all complex structures generated by the LibDock module, the binding orientation and conformation of ligand compounds similar to those of ouabain were selected. The distances of intermolecular hydrogen bonds (from proton to acceptor) were set as less than 2.5 Å.

## Additional Information

**Accession code**: Crystal data of compound **5β** in standard CIF format have been deposited in the Cambridge Crystal Data Centre with accession code CCDC 1406923.

**How to cite this article**: Tang, H.-J. *et al*. Novel stereoselective bufadienolides reveal new insights into the requirements for Na^+^, K^+^-ATPase inhibition by cardiotonic steroids. *Sci. Rep*. **6**, 29155; doi: 10.1038/srep29155 (2016).

## Supplementary Material

Supplementary Information

## Figures and Tables

**Figure 2:**
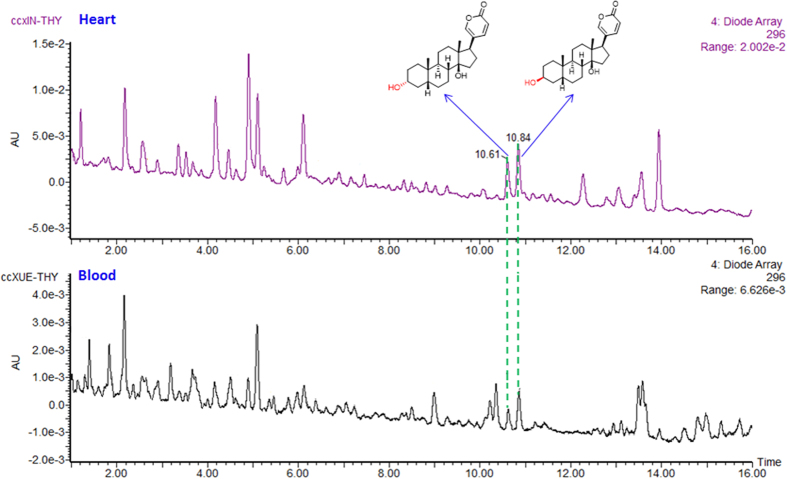
Detection of both bufalin (1β) and 3α-hydroxybufalin (1α) in the heart of and blood of *Bufo bufo gargarizansl* by UPLC analysis. Both compounds were confirmed by comparison of the retention time, online UV spectra and Mass spectra with those of the standards.

**Table 1:** Summary of inhibition data.

Compound	K_Diss,high_ (μM)	K_Diss,low_ (μM)	K_i_^a^	K_C_^b^
**1α**	13.6 ± 0.7 (85%)	805 ± 434 (15%)	77	5.67
**1β**	0.25 ± 0.02 (90%)	115 ± 63 (10%)	1.5	9.0
**4α**	7.26 ± 0.16 (90%)	524 ± 153 (10%)	65	9.0
**4β**	<0.05 ± 0.01 (86%)	>1 mM (13.5%)	0.31	6.14
**5α**	39.2 ± 1.5 (98%)	% too small	1920	49
**5β**	0.085 ± 0.01 (76%)	67.6 ± 38 (22%)	0.26	3.17
**6α**	22.7 ± 1.56 (65%)	242 ± 35 (33%)	42.2	1.86
**6β**	3.56 ± 0.60 (64%)	93.5 ± 67 (32%)	6.3	1.78

The percentages of high- and low-affinity components of the inhibition curves are shown in parentheses. Note: ^a^dissociation constant in μM; ^b^equilibrium constant.

**Table 2:** ^13^C chemical shifts (ppm) of the glucose moiety of 5α and 5β.

Position	5α			5β		
	Solution state	Solid state	NKA-bound	Solution state	Solid state^1)^	NKA-bound
C1’	89.7	90.5	ND^2)^	89.6	91.5	87.3
C2’	70.5	70.0	ND	70.6	74.0	67.0
C3’	78.8	80.0	ND	78.9	82.5, 80.0	76.1
C4’	69.7	67.4	ND	70.3	70.5	67.5
C5’	78.4	79.5	ND	78.4	79.5, 78.4	79.9
C6’	61.3	61.5	ND	61.4	62.5	62.0

Solid-state values were obtained from DARR ^13^C SSNMR spectra of **5α** and **5β** in the solid crystalline state (SI, Figure S11-2). The doubling of some resonances for **5β** in the solid-state is consistent with two molecules in the crystallographic asymmetric unit (i.e., SI, Figure S7-4). NKA bound values were estimated by peak fitting to the one-dimensional spectrum (Fig. 5A,B) and tentative assignments are from the DARR spectrum in Figure S11-2). Note: ^1)^two conformations; ^2)^ND means not detected.
