# Supplementary material for: Novel stereoselective bufadienolides reveal new insights into the requirements for Na+, K+-ATPase inhibition by cardiotonic steroids
Source: Sci Rep. 2016 Jul 5;6:29155. doi: 10.1038/srep29155 (PMC4932606; doi:10.1038/srep29155)
Supplement: Supplementary Information [file srep29155-s1.pdf]

Novel stereoselective bufadienolides reveal new insights into the requirements for Na^+,K^+-ATPase inhibition by cardiotonic steroids

Hong-Jin Tang[1†], Li-Jun Ruan[1†], Hai-Yan Tian[1], Guang-Ping Liang[1], Wen-Cai Ye[1], Eleri Hughes[2], Mikael Esmann[3], Natalya U. Fedosova[3], Tse-Yu Chung[4], Jason T.C. Tzen[4], Ren-Wang Jiang[1*], David A. Middleton[2*]

[1]College of Pharmacy, Jinan University, Guangzhou city, Guangdong province, 510632, P. R. China, [2]Department of Chemistry, University of Lancaster, Lancaster LA1 4YB, U.K, [3]Department of Biomedicine, Aarhus University, DK-8000, Aarhus, Denmark, [4]Graduate Institute of Biotechnology, National Chung-Hsing University, Taichung 40227, Taiwan, China.

[†] These authors contributed equally to this work.

Correspondence and requests for materials should be addressed to R.W.J. (email: rwjiang2008@126.com) or D. A. M (d.middleton@lancaster.ac.uk)

Outline of the supporting information

Methods

1. General methods.
2. Isolation of the starting material bufalin (**1β**).
3. Synthesis of bufalone (**2**).
4. Synthesis of 3*R*-bufalin(**1α**)
5. Synthesis of bufalone oximes (**3a/b**).
6. Synthesis of aglycones **4α** and **4β**.
7. Synthesis of glycoside **5α**.
8. Synthesis of glycoside **5β**.
9. Synthesis of 3-trifluoromethyl derivatives **6α** and **6β.**
10. X-ray analysis of **5β.**
11. Preparation and activity assay of Na^+,K^+-ATPase.
12. Details of the bufadenolide inhibition experiments
13. Details of the kinetic analysis of inhibition.
14. Sample preparation for solid-state NMR

Figures

Fig. S1-1 ESI-MS spectra confirming the presence of **1α** and **1β** in the two peak fractions shown in Figure 1 of the main text.

Fig.S1-2 UPLC analysis of the standards of 3α-hydroxybufalin (**1α**) and bufalin (**1β**)

Fig. S1-3 Detection of both bufalin (**1β**) and arenobufagin and cinobufagin in the venom of *Bufo bufo gargarizans* by UPLC analysis.

Fig. S2-1 ESI-MS spectrum of bufalin **(1β).**

Fig. S2-2 ^{1}H NMR spectrum of bufalin **(1β).**

Fig. S2-3 ^{13}C NMR spectrum of bufalin **(1β).**

Fig. S3-1 ESI-MS spectrum of 3*R*-bufalin **(1α).**

Fig. S3-2 ^{1}H NMR spectrum of 3*R*-bufalin **(1α).**

Fig. S3-3 ^{13}C NMR spectrum of 3*R*-bufalin **(1α).**

Fig. S3-4 NOESY spectrum of 3*R*-bufalin **(1α)**

Fig. S4-1 ESI-MS of **4α.**

Fig. S4-2 ^{1}H NMR spectrum of **4α.**

Fig. S4-3 ^{13}C NMR spectrum of **4α.**

Fig. S5-1 ESI-MS of **4β.**

Fig. S5-2 ^{1}H NMR spectrum of **4β.**

Fig. S5-3 ^{13}C NMR spectrum of **4β.**

Fig. S6-1 ESI-MS of **5α.**

Fig. S6-2 ^{1}H NMR spectrum of **5α.**

Fig. S6-3 ^{13}C NMR spectrum of **5α.**

Fig. S6-4 NOESY spectrum of 5α

Fig. S7-1 ESI-MS of **5β.**

Fig. S7-2 ^{1}H NMR spectrum of **5β.**

Fig. S7-3 ^{13}C NMR spectrum of **5β.**

Fig. S7-4 X-ray structure of **5β** showing two molecules in the asymmetric unit.

Fig. S8-1 HRESI-MS of **6α.**

Fig. S8-2 ^{1}H-NMR of **6α.**

Fig. S8-3 ^{13}C-NMR of **6α.**

Fig. S8-4 ^{19}F NMR of **6α.**

Fig. S8-5 NOESY spectrum of **6α.**

Fig. S9-1 HRESI-MS of **6β.**

Fig. S9-2 ^{1}H-NMR of **6β.**

Fig. S9-3 ^{13}C-NMR of **6β.**

Fig. S9-4 ^{19}F NMR of **6β.**

Fig. S9-5 NOESY spectrum of **6β.**

Fig. S10 Expansion of the inhibition curves in Figure 4 of the main text.

Fig. S11-1 ^{13}C CPMAS SNMR spectrum of NKA and **5β** in the presence of ouabain.

Fig. S11-2 ^{13}C DARR solid-state NMR spectrum of the NKA-**5β** complex.

Tabulated data

Detailed hydrogen bonds in compounds **1α**,**1β, 4α, 4β**, **5α** and **5β**

1. General procedures

Solution-state NMR spectra were obtained on a Bruker AV-400 spectrometer. ESI-MS and HRESI-MS spectra were obtained on a Finnigan LCQ Advantage Max ion trap mass spectrometer and an Agilent 6210 ESI/TOF mass spectrometer, respectively. Silica gel for column chromatography (200-300 mesh) was produced by Qingdao Marine Chemical Industrials. Precoated silica gel GF_{254} plates (Qingdao Marine Chemical Plant, Qingdao, P. R. China) were used for TLC analysis. Preparative HPLC was performed on a Varian Prostar system equipped with a preparative Cosmosil C_{18} column (5μm, 20×250 mm) column. D-glucose (U-13C_6, 99%) was purchased from Cambridge isotope laboratories (MA, USA). Other reagents for chemical reactions were purchased from Sigma-Aldrich Inc

2. Isolation of the starting material bufalin (1β)

The dried and powdered venom (1.0 kg) of *Bufo bufo Gargarizans* was extracted by 95% ethanol under ultrasonic condition (30 min, 40 °C) four times (each half hour). The combined extract was concentrated under reduced pressure to provide a residue (280 g), which was subsequently partitioned between methylene dichloride (CH_2Cl_2) and water. The CH_2Cl_2 solution was evaporated to give a residue (130 g), which was then subjected to silica gel (200 – 300 mesh) chromatography, eluted with gradient cyclohexane–acetone to give five fractions (Fr.1–5). Fr. 3 was separated by silica gel chromatography again eluted with cyclohexane–ethyl acetate gradients gradient (20:80 to 90:10) to afford bufalin (4.65 g).

Bufalin: colorless crystal (CH_3OH), ESI-MS *m/z* 387 $[M+H]^+$ and 795 $[2M+Na]^+$; ^{1}H NMR ($CDCl_3$, 300 MHz): δ_H 7.84 (1H, dd, *J* = 9.7, 2.5 Hz), 7.23 (1H, d, *J* = 2.5 Hz), 6.26 (1H, d, *J* = 9.7 Hz), 4.14 (1H, br s), 2.45 (1H, dd, *J* = 9.6, 6.5 Hz), 0.70 (3H, s), 0.95 (3H, s); ^{13}C NMR ($CDCl_3$, 75 MHz): δ_C 16.5, 21.4, 21.4, 23.7, 26.5, 27.9, 28.7, 29.6, 32.7, 33.3, 35.4, 35.7, 36.0, 40.9), 42.4, 48.3, 51.3, 66.8, 85.3, 115.4, 122.6, 146.7, 148.5, 162.3.

3. Synthesis of bufalone (2). Bufalin (4.13g, 10.7mmol) was dissolved in

CH_2Cl_2 (35 ml) in a round bottom flask. Pyridinium chloride hydrochloride (PCC) (4.60g, 21.4mmol) was added, stirring constantly to completely dissolve. The solution was stirred for two hours at room temperature. The solvent was removed under reduced pressure. The crude reaction mixture was purified by silica gel column chromatography, eluting with 1:1 hexane/EtOAc. Bufalone was obtained as a white powder (3.9g, 95% yield, R_f = 0.35 hexane/EtOAc 1:1). ^1H NMR ($CDCl_3$, 300MHz) δ7.83 (*dd*, 1H, *J* = 9.6, 2.6Hz), 7.22 (*d*, 1H, *J* = 2.6Hz), 6.23 (*d*, 1H, *J* = 9.6Hz), 2.62 (1H, *t*, *J* = 10.7Hz), 2.46 (*dd*, 1H, *J* = 9.4, 6.1 Hz, H-17), 2.33 (*m*, 1H), 2.19 (*m*, 2H), 2.19-1.20 (*m*, 19H), 0.983 (*s*, 3H), 0.703 (*s*, 3H). ^{13}C ($CDCl_3$, 75MHz) δ212.9, 162.5, 148.6, 146.9, 122.7, 115.3, 85.0, 51.2, 48.4, 43.7, 42.15, 42.13, 40.7, 37.1, 36.8, 36.7, 35.2, 32.7, 28.7, 26.6, 22.6, 21.5, 21.0, 16.6. ESI-MS m/z for $C_{24}H_{33}O_4$: 385.5 $[M+H]^+$, 407.4$[M+Na]^+$, 791.4$[2M+H]^+$, 823.3 $[2M+Na]^+$; HR-ESIMS m/z for $C_{24}H_{32}NaO_4$ ($[M+Na]^+$) 407.2194, cacl. 407.2198.

4. Synthesis of 3*R*-bufalin (1α)

Bufalone (76.8mg, 0.2mmol) was dissolved in anhydrous tetrahydrofuan (5 ml) in a round bottom flask. Sodium borohydride ($NaBH_4$) was added, stirring constantly to completely dissolve. The solution was stirred for one hour at room temperature. Then 5ml water was added slowly to the reaction solution. Ethyl acetate (5ml × 2) was used to extract the mixture and the solvent was removed under reduced pressure. The crude reaction mixture was purified by preparative HPLC, eluting with 45% acetonitrile to yield **3*R*-bufalin** (58.0 mg, yield 75%).

3*R*-bufalin: colorless crystal (CH_3OH), ESI-MS *m/z* 387 $[M+H]^+$ and 795 $[2M+Na]^+$; ^1H NMR ($CDCl_3$ 300 MHz)：7.86 (1H, dd, *J* = 9.6, 2.2 Hz), 7.24 (1H, d, *J* = 2.2 Hz), 6.28 (1H, d, *J* = 9.6 Hz), 3.65(1H, m)，2.48 (1H, m)，0.92 (3H, s)，0.71 (3H, s); ^{13}C NMR ($CDCl_3$ 75 MHz): δ_C 16.5, 21.2, 21.5, 23.2, 27.0, 28.7, 30.5, 32.7, 34.8, 35.0, 36.2, 36.4, 40.8, 41.5, 42.5, 48.3, 51.2, 71.6, 85.3, 115.3, 122.7 146.8, 148.5, 162.4.

5. Synthesis of bufalone oximes (3a/b). Bufalone (3.9g, 10.2mmol) was dissolved in CH_3OH (28 ml) in a 50 ml round bottom flask. Methoxylamine

hydrochloride (2.56g, 30.6mmol) was added, followed by addition of pyridine (4.88ml, 60.7mmol). The mixed solution was stirred for 1.5 hours at room temperature. The solvent was removed in *vacuo* and the resulting residue was dissolved in CH_2Cl_2. It was washed with 1mol/L HCl, then brine, and extracted four times with CH_2Cl_2 (20ml). The organic layer was dried over Na_2SO_4, filtered, and concentrated. The desired product of mixed oxime diastereomers (**3a/b**) was obtained as a white solid (3.78g, 90% yield, R_f = 0.57 and 0.63 hexane/EtOAc 1:1), and used without further purification. ^{1}H NMR ($CDCl_3$, 300MHz) δ7.77 (dd, H, *J* = 9.7, 2.6Hz), 7.15 (d, 1H, *J* = 2.6Hz), 6.17 (d, 1H, *J* = 9.7Hz), 3.73 (s, 3H), 2.76 (dd, 1H, *J* = 15.4, 4.4Hz), 2.39 (m, 1H), 2.03, 1.70 (m, 2H), 2.19, 1.74 (m, 2H), 2.15, 1.85 (m, 2H), 1.89, 1.23 (m, 2H), 1.55, 1.25 (m, 2H), 2.46 (m, 1H), 1.65 (m, 1H), 1.54 (m, 1H), 0.87 (s, 3H), 0.62 (s, 3H). ^{13}C NMR ($CDCl_3$, 75MHz) δ162.4, 160.3, 148.4, 146.8, 122.7, 115.1, 85.0, 60.9, 51.0, 48.2, 42.1, 41.6, 40.5, 36.6, 36.5, 35.4, 32.6, 28.6, 26.7, 26.4, 25.4, 22.9, 21.3, 20.9, 16.4. ESI-MS m/z for $C_{25}H_{35}NO_4$: 414.5 $[M+H]^+$, 436.3 $[M+Na]^+$, 849.5 $[2M+Na]^+$; HRMS (ESI) m/z for $C_{25}H_{35}NO_4Na$ ($[M+Na]^+$) 436.2468, calculated 436.2463.

6. Synthesis of aglycones 4α and 4β. Bufalone oximes (**3a/b**) (400mg, 0.969mmol) was dissolved in mixed solvents dioxane/ethanol 2:1 (4.5 ml) in a 15 ml reaction tube, and then cooled to 0℃. After adding borane *tert*-butylamine complex (278.10mg, 3.198mmol), 10% aqueous hydrochloric acid solution (2.61 ml) was added dropwise over two minutes. The mixed solution was stirred at 0℃ for 3.5 hours. After this time, the reaction mixture was concentrated under *vacuo*, and CH_2Cl_2 (20ml) was added. The mixture was washed with saturated aqueous $NaHCO_3$ (10ml), and then separated between saturated brine and CH_2Cl_2. The crude CH_2Cl_2 extract was separated by silica gel column chromatography. Elution of the column with hexane/EtOAc 4:1 afforded aglycone **4β** (88.5mg, 22% yield, R_f = 0.35 hexane/EtOAc 1:1), and then elution with 100% EtOAc afforded **4α** (175mg, 43% yield, R_f =0.25 hexane/EtOAc 1:1).

Aglycone **4α** was obtained as white foam. ^{1}H NMR ($CDCl_3$, 300MHz) δ7.83 (dd, 1H, *J* = 9.7, 2.5Hz), 7.21 (d, 1H, *J* = 2.5Hz), 6.24 (d, 1H, *J* = 9.7Hz), 3.54 (s,

3H), 2.91 (m, 1H), 2.43 (m, 1H), 2.17,1.71 (m, 2H), 2.06, 1.66 (m, 2H), 1.80, 1.02 (m, 2H), 1.83, 1.36 (m, 2H), 1.47, 1.31 (m, 2H), 1.71, 1.27 (m, 2H), 1.60, 1.17 (m, 2H), 1.54, 1.45 (m, 2H), 1.38, 1.16 (m, 2H), 1.63 (m, 1H), 1.51 (m, 1H), 1.40 (m, 1H), 0.91 (s, 3H), 0.67 (s, 3H). ^{13}C NMR ($CDCl_3$, 75MHz) δ162.4, 148.4, 146.8, 122.7, 115.2, 85.3, 62.6, 60.3, 51.1, 48.2, 42.5, 41.4, 40.8, 36.4, 35.4, 35.1, 32.7, 31.0, 28.6, 27.1, 25.3, 23.4, 21.5, 21.1, 16.4. ESI-MS m/z for $C_{25}H_{37}NO_4$: 416.3 $[M+H]^+$, 438.3 $[M+Na]^+$, 831.5 $[2M+H]^+$, 853.4 $[2M+Na]^+$; HRMS (ESI) m/z for $C_{25}H_{37}NO_4Na$ ($[M+Na]^+$) 438.2625, calculated 438.2620.

Aglycone **4β** was also obtained as a white powder. ^{1}H NMR ($CDCl_3$, 300MHz) δ7.84 (dd, 1H, *J* = 9.7, 2.5Hz), 7.21 (d, 1H, *J* = 2.5Hz), 6.24 (d, 1H, *J* = 9.7Hz), 3.53 (s, 3H), 3.24 (s, 1H), 2.46 (m, 1H), 2.17,1.70 (m, 2H), 2.04, 1.67 (m, 2H), 1.44, 1.33 (m, 2H), 1.38, 1.23 (m, 2H), 1.45, 1.35 (m, 2H), 1.69, 1.15 (m, 2H), 1.86, 1.27 (m, 2H), 1.81, 1.23 (m, 2H), 1.84, 1.35 (m, 2H), 1.62 (m, 1H), 1.51 (m, 1H), 1.43 (m, 1H), 0.90 (s, 3H), 0.67 (s, 3H). ^{13}C NMR ($CDCl_3$, 75MHz) δ162.3, 148.4, 146.8, 122.7, 115.1, 85.3, 62.3, 54.9, 51.1, 48.2, 42.3, 40.8, 36.5, 35.7, 35.4, 32.7, 30.2, 28.6, 28.5, 26.5, 23.7, 22.7, 21.2, 21.1, 16.4. ESI-MS m/z for $C_{25}H_{37}NO_4$: 416.4 $[M+H]^+$, 438.4 $[M+Na]^+$, 831.4 $[2M+H]^+$, 853.3 $[2M+Na]^+$; HRMS (ESI) *m/z* for $C_{25}H_{37}NO_4Na$ ($[M+Na]^+$) 438.2623, calculated 438.2620.

7. Synthesis of glycoside 5α. Aglycone **4α** (35mg, 0.084mmol) was added to a 5 ml micro reaction tube and dissolved in mixed solvents DMF/AcOH (3:1, 1339μl). After adding D-glucose (U-13C6, 99%, 22.8mg, 0.127mmol), the mixture was stirred at 40℃ for two days. Then the solvents were removed in *vacuo*, and the crude material was dissolved in a minimal volume of methanol. The final purified neoglycoside **5α** was obtained as a white powder (23.36mg, 48% yield, R_f = 0.48 hexane/EtOAc 3:7) by preparative high-performance liquid chromatography (chromatographic conditions: flow rate was 8ml/min, detection wavelength was 296nm, and the retention time was 15.02 min with 45% acetonitrile/water as the mobile phase).

^{1}H NMR (Pyridine-d_5, 300MHz) δ 8.33 (dd, 1H, *J* = 9.7, 2.5Hz), 7.56 (d, 1H, *J* = 2.5Hz), 6.44 (d, 1H, *J* = 9.7Hz), 4.90 (d, 1H, *J* = 8.7Hz), 4.57 (m, 1H), 4.39 (m,

1H), 4.31 (m, 1H), 3.98 (m, 1H), 4.63, 4.47 (m, 2H), 4.06 (s, 3H), 3.66 (m, 1H), 2.27, 2.58 (m, 1H), 2.07 (m, 2H), 2.26 (m, 2H), 1.93 (m, 2H), 1.90, 1.60 (m, 2H), 1.02 (m, 2H), 0.97 (s, 3H), 0.89 (s, 3H). ^{13}C NMR (DMSO-d_6, 75 MHz) δ 161.4, 149.4, 147.4, 122.7, 114.2, 89.7 (d, J = 47.6Hz), 83.9, 78.8, 78.4, 70.4, 69.9, 62.8, 61.3, 60.9, 50.1, 48.0, 41.37, 41.34, 39.9, 35.7, 35.4, 34.6, 32.1, 31.2, 28.4, 27.2, 24.1, 23.3, 21.3, 20.8, 16.6. ESI-MS *m/z* $^{12}C_{25}{}^{13}C_6H_{47}NO_9$:584.5 $[M+H]^+$, 606.5 $[M+Na]^+$, 1189.5 $[2M+Na]^+$.

HR-ESIMS for the natural abundance **5α** (synthesized with the natural abundance glucose) *m/z* $C_{31}H_{47}NO_9Na$ ($[M+Na]^+$) 600.3145, cacl. 600.3148.

8. Synthesis of glycoside 5β. Aglycone **4β** (33mg, 0.795mmol) was added to a 5ml micro reaction tube and dissolved in mixed solvents DMF/AcOH (3:1, 1204μl). After adding D-glucose (U-13C6, 99%, 21.5mg, 0.119mmol), the mixture was stirred at 40℃ for two days. Then the solvents were removed *in vacuo*, and then the crude material was dissolved in a minimal volume of methanol. The desired purified neoglycoside **5β** was obtained as a crystalline solid with a yield 42%, R_f = 0.59 hexane/EtOAc 3:7) by preparative high-performance liquid chromatography (chromatographic conditions: flow rate was 8ml/min, detection wavelength was 296nm, and the retention time was 17.38min with 45% acetonitrile/water as the mobile phase).

^1H NMR (Pyridine-d_5, 300MHz) δ 8.23 (dd, 1H, J = 9.7, 2.5Hz), 7.48 (d, 1H, J = 2.5Hz), 6.32 (d, 1H, J = 9.7Hz), 4.69 (d, 1H, J = 8.7Hz), 4.47 (m, 1H), 4.26 (m, 1H), 4.25 (m, 1H), 4.54, 4.36 (m, 2H), 3.98 (s, 3H), 3.88 (brs, 1H), 2.20, 1.85 (m, 2H), 2.02, 1.86 (m, 2H), 2.00, 1.58 (m, 2H), 1.43, 1.33 (m, 2H), 1.35, 1.13 (m, 2H), 1.63, 1.07 (m, 2H), 2.49 (m, 1H), 2.02 (m, 2H), 1.87 (m, 2H), 0.87 (s, 3H), 0.82 (s, 3H).^{13}C NMR (DMSO-d_6, 100 MHz) δ 161.4, 149.2, 147.4, 122.8, 114.2, 89.6 (d, J = 47.9Hz), 83.4, 78.9, 78.4, 70.6, 70.0, 62.7, 61.4, 56.1, 50.1, 48.1, 41.4, 40.4, 36.1, 35.4, 35.3, 32.0, 30.4, 29.0, 28.5, 27.0, 24.5, 23.8, 21.2, 20.8, 16.7. ESI-MS m/z $^{12}C_{25}{}^{13}C_6H_{47}NO_9$: 584.5 $[M+H]^+$, 606.5 $[M+Na]^+$, 1189.5 $[2M+Na]^+$.

HR-ESIMS for the natural abundance **5β** (synthesized with the natural abundance glucose) *m/z* $C_{31}H_{47}NO_9Na$ ($[M+Na]^+$) 600.3143, cacl. 600.3148.

9. Synthesis of 3-trifluoromethyl derivatives 6α and 6β.

Bufalone (**2**, 0.2 mmol) was dissolved in anhydrous THF (5 ml), then catalytic amount of *n*-tetra butylammonium fluoride (approx. 6 mg) was added. The mixture was protected against the moisture. Trimethyl(trifluorometylhyl)silane (1.0 mmol) was added in dropwise. The mixture was stirred for 1.5 h at room temperature. After this time, 30 mL H_2O was added and extracted with ethyl acetate. The organic layer was washed with saturated NaCl solution, and dried over anhydrous sodium sulfate. Then the solvent was removed to afford a yellow residue, which was the trimethylsilane protected product.

The yellow residue was dissolved in methanol (5 ml), and CsF (5eq.) was added. The mixture was refluxed for 40 hour. After the reaction, methanol was removed under vacuum. The residue was dissolved in ethyl acetate and washed by saturated NaCl solution. Then the organic layer was dried and the solvent was removed under vacuum. Finally the residue was purified by preparative HPLC to afford **6α** (R_t= 10.1min, 4.9 mg, yield 10.7%) and **6β** (R_t= 11.3min, 12.4 mg, yield 27.3%) (column: cosmosil 10×250mm, 5μm, C18-MS-II, mobile phase: isocratic 75% methanol in water).

Compound **6α** was obtained as white powder. ESI-MS *m/z* $C_{25}C_{33}O_4F_3$: 455.2 $[M+H]^+$; HRESI-MS *m/z* 455.2403 $[M+H]^+$, 477.2224 $[M+Na]^+$; ^1H NMR (DMSO, 300 MHz)：δ_H 7.91 (1H, dd, J = 9.0, 3.0 Hz), 7.51 (1H, d, J = 2.5 Hz)，6.27 (1H, d, J = 9.0 Hz), 5.62 (3-OH), 4.19 (14-OH), 0.85 (3H, s), 0.58 (3H, s); ^{13}C NMR (DMSO, 75 MHz): δ_C 162.6, 150.5, 148.6, 123.9, 115.5, 84.5, 72.1 51.3, 49.3, 42.3, 41.4, 41.3, 40.2, 39.9, 37.4, 36.2, 35.6, 33.1, 30.9, 30.8, 29.7, 27.3, 24.5, 22.3, 17.9; ^{19}F NMR ($CDCl_3$, 282.4 MHz) δ -82.98.

Compound **6β** was obtained as white powder. ESI-MS *m/z* $C_{25}C_{33}O_4F_3$: 455.3 $[M+H]^+$; HRESI-MS *m/z* 455.2396 $[M+H]^+$, 477.2209 $[M+Na]^+$; δ_H 7.93 (1H, dd, J = 9.0, 3.0 Hz), 7.52 (1H, d, J = 2.5 Hz)，6.28 (1H, d, J = 9.0 Hz), 5.72 (3-OH), 4.19 (14-OH), 0.86 (3H, s), 0.60 (3H, s); ^{13}C NMR (DMSO, 75 MHz): δ_C 162.6, 150.5, 148.6, 123.9, 115.5, 84.6, 73.0, 51.4, 49.3, 42.4, 41.6, 42.3, 39.5, 37.1, 35.3, 34.8, 33.5, 33.3, 29.6, 29.0, 27.5, 24.2, 22.3, 21.9, 17.9; ^{19}F NMR ($CDCl_3$,

282.4 MHz) δ -77.08.

10. X-ray analysis of 5β

X-ray diffraction data were collected on an Gemini S ultra spharrie CCD diffractometer using graphite monochromated radiation (λ = 1.54178 Å) at room temperature. The crystal structures were elucidated by direct methods using SHELXS-97 and refined by full-matrix least-squares method on F^2 using SHELXS-97. In the structure refinement, non-hydrogen atoms were refined anisotropically. Hydrogen atoms bonded to carbons were placed at their geometrically ideal positions. Hydrogen atoms bonded to oxygen were located by employing the difference Fourier method and were included in the calculation of structure factors with isotropic temperature factors.

Colorless crystals was obtained from methanol solution, triclinic, *P*1, a = 10.4551(7), b = 10.4670(6), c = 15.8564(10) Å, α= 80.728(5), β= 73.259(6), γ= 65.749(6)°, V = 1513.22(16) $Å^3$, Z = 2, d_x = 1.268 Mg/m^3, $\mu(CuK\alpha)$ = 0.757 mm^{-1}, F (000) = 624. 5453 unique reflections were collected to θ_{max} = 62.86°, in which 4730 reflections were observed [$F^2 > 4\sigma(F^2)$]. The final R = 0.1248, S = 1.406 and CCDC 1406923.

11. Preparation and activity assay of Na^+,K^+-ATPase

Na^+,K^+-ATPase from pig kidney microsomal membranes was purified by differential centrifugation.[27] The specific Na,K-ATPase activity was determined from the difference in the amount of phosphate released in the absence and presence of 1 mM ouabain in a solution containing 130 mM NaCl, 20 mM KCl, 4 mM $MgCl_2$ and 3 mM ATP. The specific activity of the enzyme preparation was approximately 30 μmol ATP hydrolysed/mg protein per min at 37°C.[27] The phosphorylation capacity is about 2.9 nmol/mg protein and is equal to the high-affinity ouabain binding capacity.[10, 27]

12. Details of the kinetic analysis of inhibition

(1) Experimental details of the bufadenolide inhibition

Stock solutions of the inhibitors, typically 10 mM, were prepared in DMSO.

The inhibitory effects of bufadienolides on NKA were determined essentially as previously reported[9, 10]. In brief, NKA is preincubated at 37° C for 2 hours in the presence of 3 mM $MgCl_2$, 3 mM Na-phosphate and 40 mM Tris (pH 7.0) with increasing concentrations of inhibitor. The residual NKA activity is subsequently determined by 40-fold dilution into a standard ATPase assay medium in triplicate, see section SI 11. For each inhibitor concentration the residual activity was determined in two or three independent experiments and the error bars in Fig. 4 indicate the standard deviation. Data in Fig. 4 are given as percent of the NKA activity in the absence of inhibitor (see legend to Fig. 4 for details).

The maximal DMSO concentration of 2.5% after dilution into the NKA incubation medium was 2.5 %. Control experiments showed that incubation with 2.5% DMSO for 2 hours at 37 ^{o}C leads to less than 10% inhibition of Na^+, K^+-ATPase activity.

(2) Detailed explanation of the kinetic analysis of inhibition

The interaction between bufalins (I) and Na^+,K^+-ATPase (E) is interpreted as a two-step process:

$$E + I \leftrightarrow E{\bullet}I \leftrightarrow E{\bullet}I^*$$

where both complexes E•I and E•I* are enzymatically inactive and in relatively slow equilibrium.

The binding of I to E is described by a dissociation constant

$$K_i = [E] \bullet [I]/[E{\bullet}I]$$

The conformational change

$$E{\bullet}I \leftrightarrow E{\bullet}I^*$$

is described by an equilibrium constant

$$K_C = [E{\bullet}I^*]/[E{\bullet}I]$$

After equilibrium is attained by preincubation of the enzyme and inhibitor, the residual Na^+,K^+-ATPase activity is measured by a 40-fold dilution into the assay medium for 2 min. It is assumed that the dilution leads to an immediate dissociation of E•I into E + I, and it is also assumed that the conformational

change E•I ↔ E•I* is slow on the 2-minutes time scale of the functional assay. These assumptions are substantiated in ref. (10).

The observed decrease in the Na^+,K^+-ATPase activity is thus an estimate of the amount of E•I* present at a given inhibitor concentration in the preincubation media, and the observed concentration dependence of inactivation is related to K_i as well as K_C.

The data are fitted by a non-linear least squares function relating the NKA activity (Activity(I)) at a given concentration of inhibitor [I] to a sum of two hyperbolic terms containing the parameters for the high-affinity as well as the low-affinity components of inhibition:

$$\text{Activity(I)} = 100\% - F_{high} * [I]/(K_{Diss,high} + [I]) - F_{low} * [I]/(K_{Diss,low} + [I])$$

The two components have magnitudes of F_{high} and F_{low} (in %). The dissociation constant derived from the curve fitting ($K_{Diss,high}$) is related to K_i and K_C through the equation $K_{Diss,high} = K_i/(K_c + 1)$.

Following the above analysis, the relative magnitude of the high- and low-affinity component is a measure of K_C. Taking compound **1α** as an example (see Table 1), we observe that 85% of activity (F_{high}) is associated with high affinity inhibitor binding (and thus F_{low} = 15% with low affinity), which gives K_C = 85%/15% = 5.67. For **1α** a dissociation constant K_i for the initial binding of inhibitor to E is $K_i = K_{Diss,high} \bullet (K_c + 1)$ = 77 µM.

The "low-affinity" component determined in the Na^+,K^+-ATPase assay reflects the sensitivity of the free enzyme (E) in the assay medium to the inhibitor carried over from the preincubation medium (and 40-fold diluted). It reflects inhibitor binding under ATP-hydrolysis conditions, which are very different from the preincubation medium with phosphate (and no ATP) present.

The eight inhibitors have widely different kinetic properties, and it is illustrative to consider three of them in detail.

Compound **5α** inactivates NKA with a single component (F_{low} = 0, Table 1) which is similar to the inactivation mode of ouabain[10]. In terms of the model

above there is virtually no E•**I** at equilibrium, all is displaced towards E•**I*** (K_c is very large, 49, see Table 1). The initial binding step is described by K_i = 1920 µM, indicating a much weaker initial binding than for compound **1α** (see above). Note that when the preincubated enzyme is diluted into the ATPase assay medium the activity determined as a function of the inhibitor concentration is proportional to the remaining free enzyme (E).

Compound **4β** inactivates the Na,K-ATPase with about 86% (=F_{high}) of the activity being very sensitive towards the inhibitor and the remaining activity is not affected by further increase in inhibitor concentration ($K_{Diss,low}$ > 1 mM, see Table 1). The simplest interpretation of this finding is that the equilibrium between E•**I** and E•**I*** is displaced 6.14 fold (= K_c) towards E•**I***, which represents 86% of the enzyme. K_i is about 0.31µM, indicating a much stronger initial binding of this compound than for compounds **1α or 5α**. Upon 40-fold dilution into the ATPase assay medium E•**I** (equal to 14%) is dissociated towards E + I, and we determine the about 14% (=F_{low}) of Na,K-ATPase activity originating from the E•**I**-complex in the preincubation medium. The E•**I*** complex dissociates extremely slowly on the 2-min time scale of the ATPase assay (see ref. 10). For the **4β** compound it is observed that there is virtually no low-affinity inactivation in the concentration ranges studied here. It seems that **4β** lacks inactivating potency in the ATPase assay medium ($K_{Diss,low}$ is very large).

Compound **5β**, on the other hand, displays a composite inactivation pattern with a high-affinity inactivation as well as an observable low-affinity inactivation (in contrast to that described above for compound **4β**). K_i is about 0.26 µM, similar to that of compound **4α.** About 22% of the ATPase activity is lost with a $K_{Diss,low}$ of about 68 µM, see Table 1. We interpret this as reflecting the inactivating potency of **5β** in the ATPase assay medium. In the preincubation situation about 22% of the enzyme is in the E•**I**-form, and in the ATPase assay medium this is dissociated towards E + I. Compound **5β** is thus potent enough to inactivate the free enzyme E in the ATPase assay medium.

In control experiments without preincubation we have observed that addition of Compound **5β** to the ATPase assay medium directly indeed inactivates the Na,K-ATPase activity and with the same potency as observed in Figure 4C (data not shown).

It should be noted that the errors in the values for $K_{Diss,low}$ in the present experiments (see Table 1) are too large to allow meaningful correlation between the low-affinity inhibitory potency and the conformation of the inhibitor.

13. Sample preparation for solid-state NMR

Na^+,K^+-ATPase membranes (13 nmol protein) were prepared as a pellet by centrifugation at 100,000× *g* for 30 min at 4°C, resuspended in 1 ml incubation medium, and incubated with 16 nmol of labeled inhibitor (**5α** and **5β**) for 60 min at 25°C. The suspension was centrifuged (100,000 × *g* at 4°C) for 30 min, and the pellet was transferred to a 4-mm external diameter zirconia MAS rotor fitted with Kel-F inserts to confine the sample to the center of the rotor. In ouabain pre-incubation experiments, the membranes were first incubated with ouabain for 1h, then the same amount of **5β** was added and further incubated for 1h.

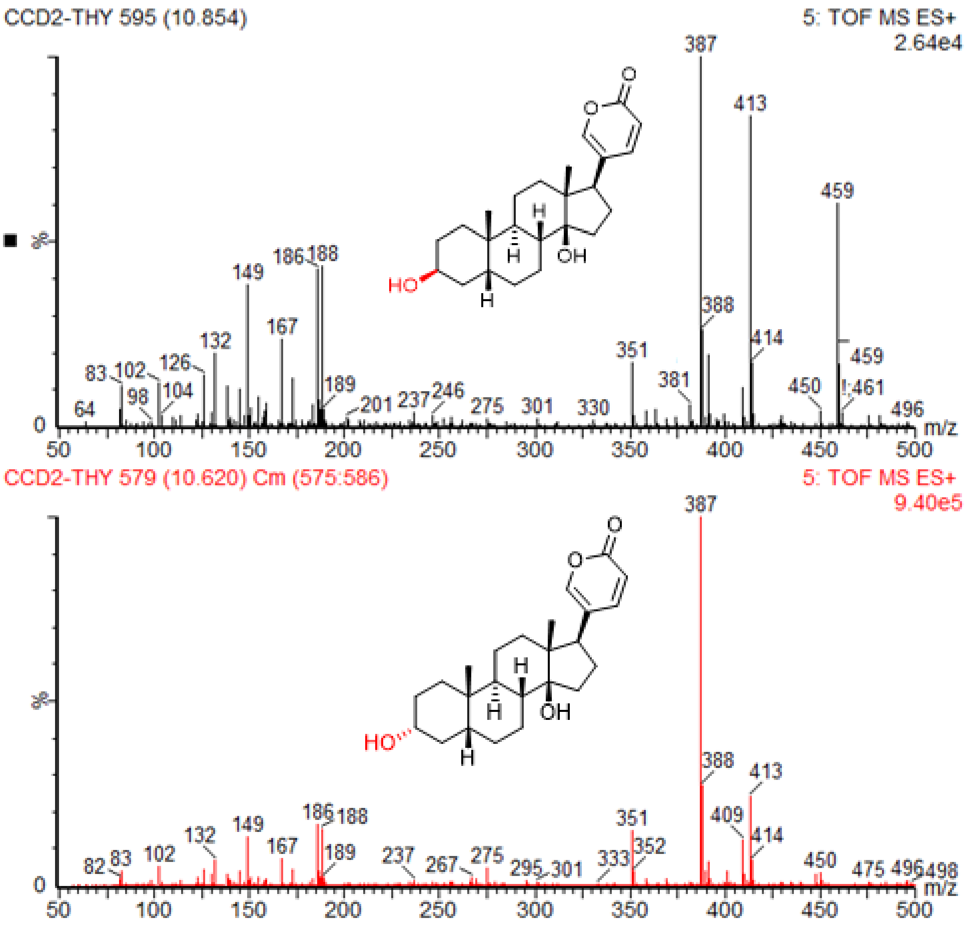

Fig. S1-1. ESI-MS of the two peaks at R_t=10.62min and R_t=10.85 min

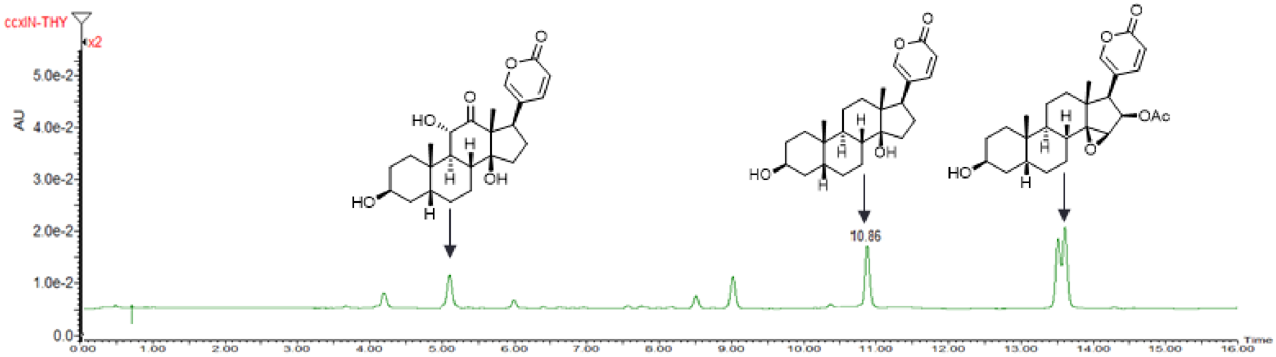

Fig. S1-2. Detection of major bufadienolides in the total bufadienolides (0.1mg/mL, injection 5.0 μL) of the venom of *Bufo bufo gargarizans* by UPLC analysis. 3α-hydroxybufalin (**1α**) was not detected in the venom, while the major bufadienolides with 3β-OH could be determined (arenobufagin R_t = 5.10 min, bufalin R_t =10.86 min, and cinobufagin R_t = 13.55 min)

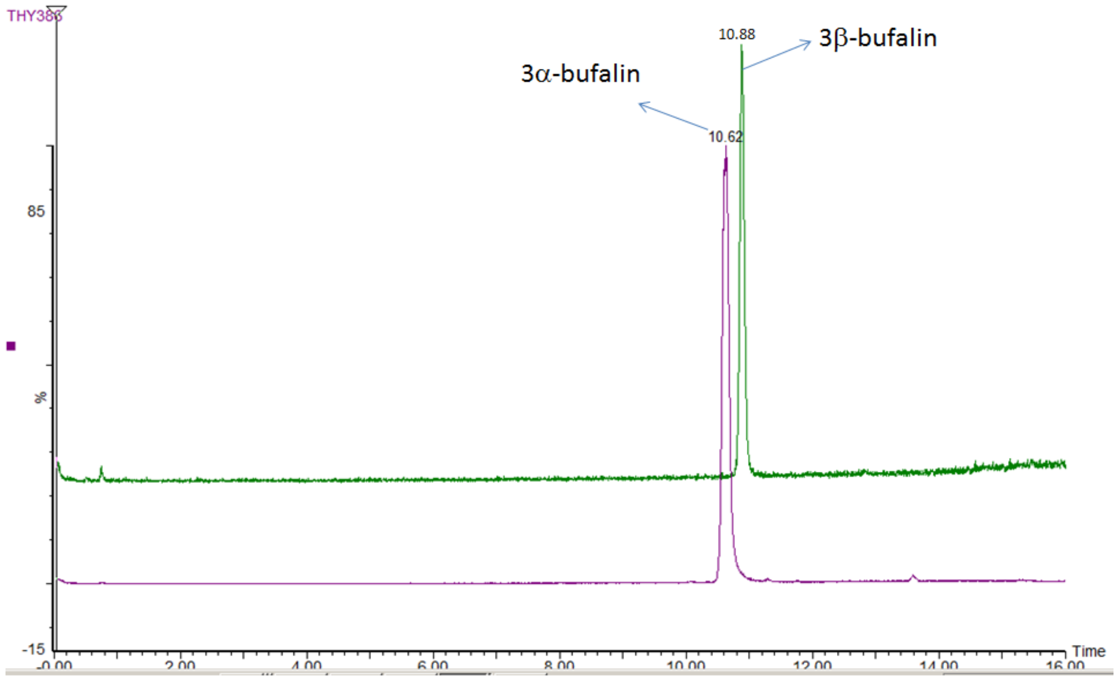

Fig.S1-3 UPLC analysis of the standards of 3α-hydroxybufalin (**1α**) and bufalin (**1β**)

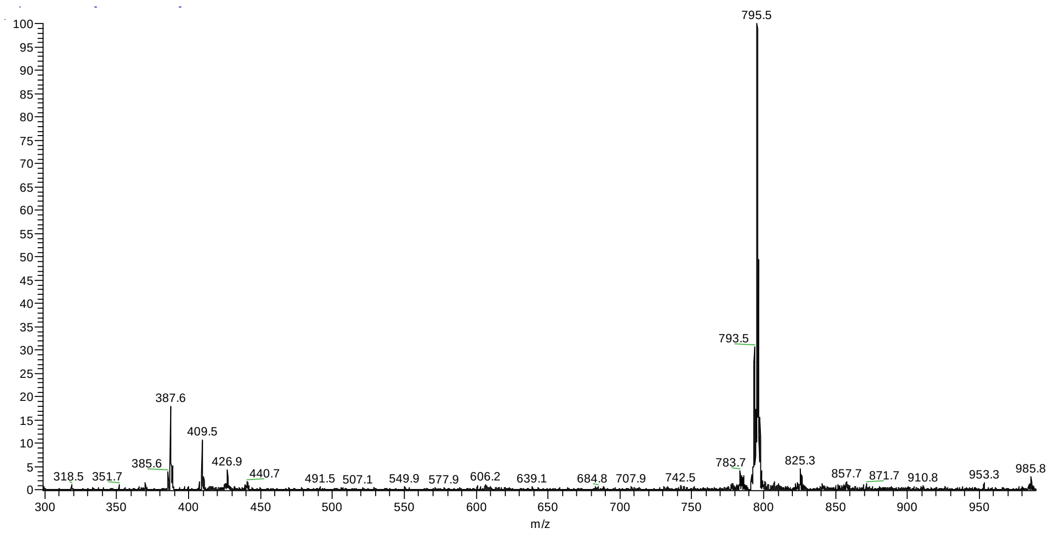

Fig.S2-1 ESI-MS spectrum of **bufalin**

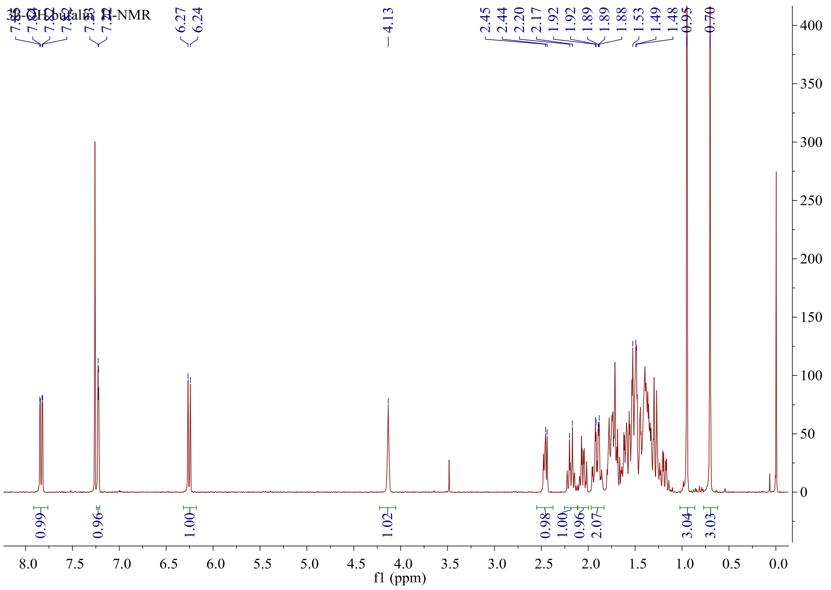

Fig. S2-2 ^{1}H NMR spectrum of **bufalin**

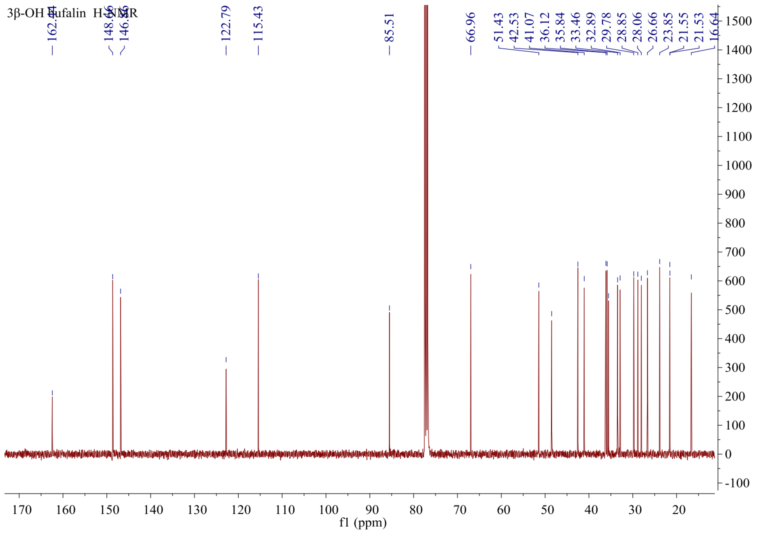

Fig. S2-3 ^{13}C NMR spectrum of **bufalin**

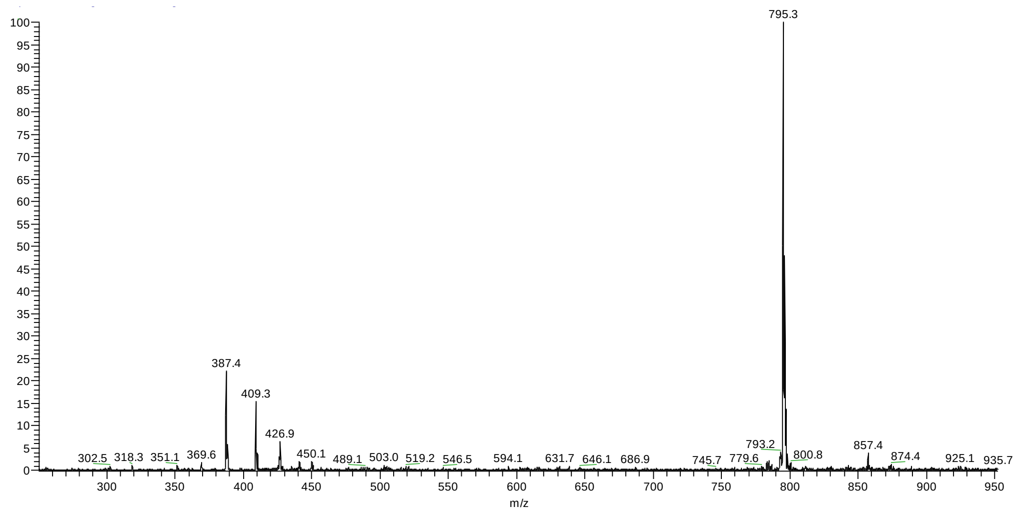

Fig. S3-1 ESI-MS spectrum of **3*R*-bufalin**

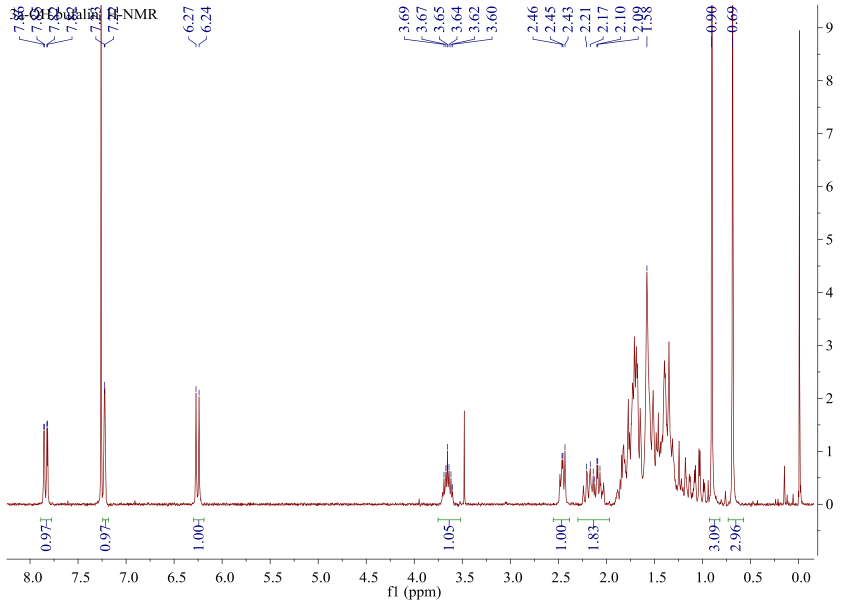

Fig. S3-2 ^{1}H NMR spectrum of **3*R*-bufalin**

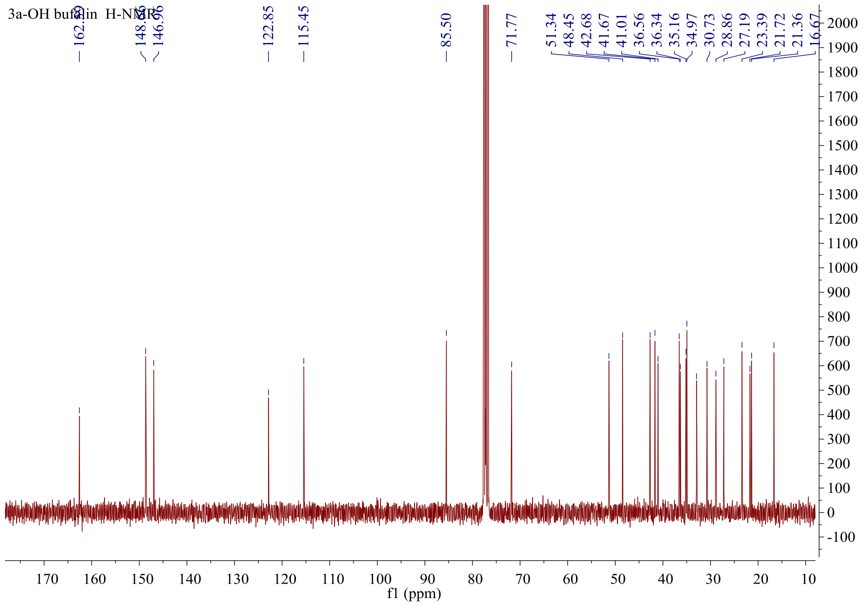

Fig. S3-3 ^{13}C NMR spectrum of **3*R*-bufalin**

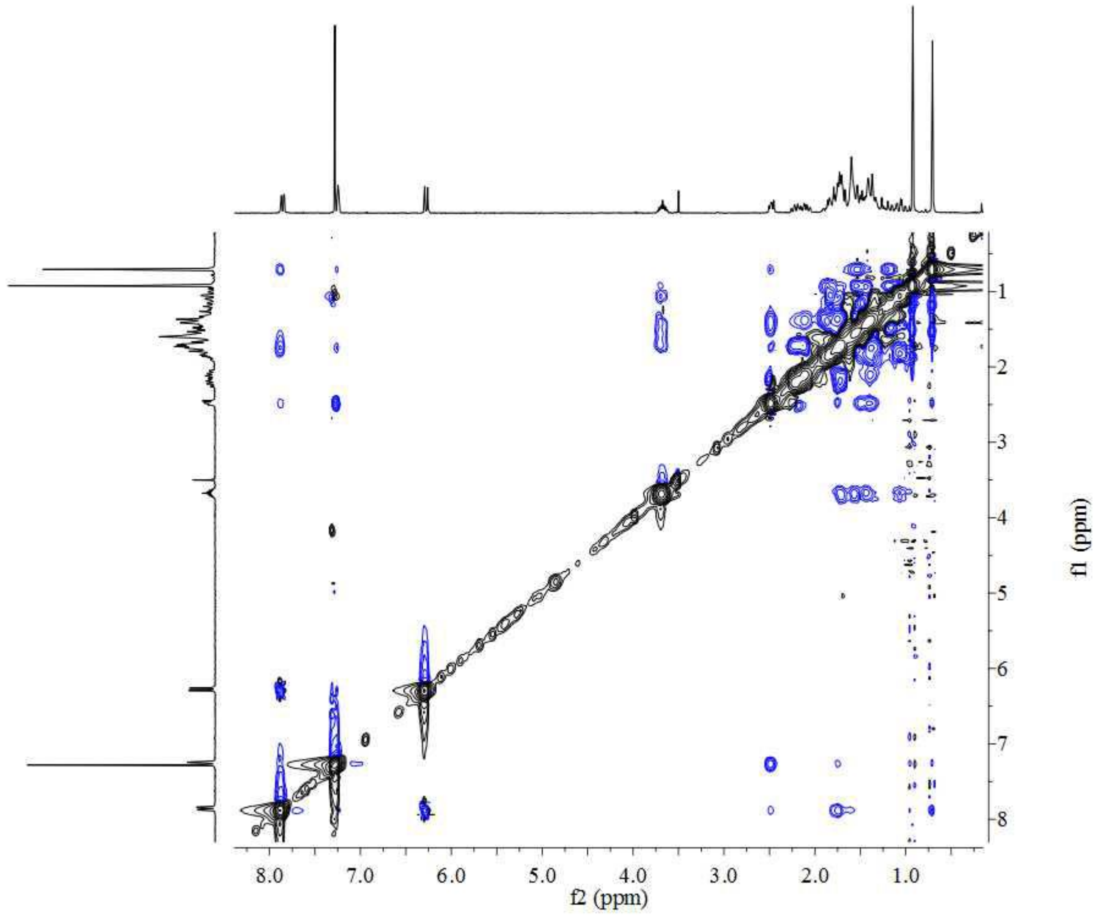

Fig. S3-4 NOESY spectrum of 3*R*-bufalin

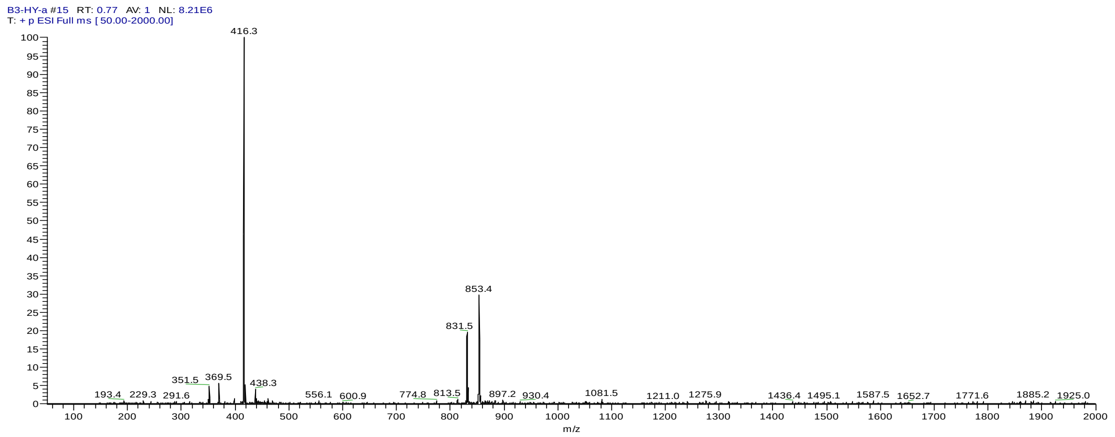

Fig. S4-1 ESI-MS of **4α**

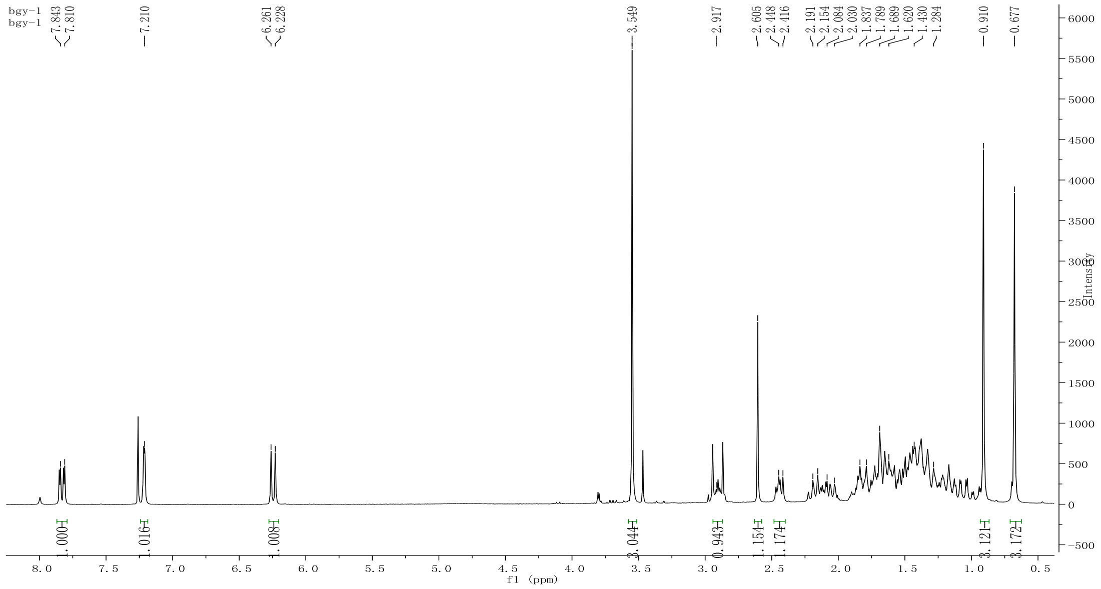

Fig. S4-2 ^{1}H NMR spectrum of **4α**

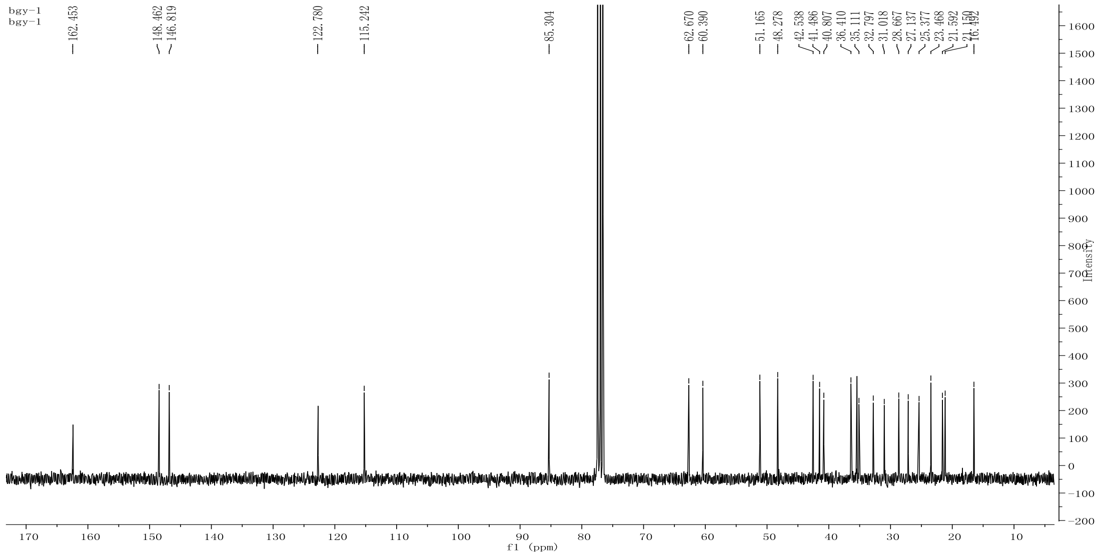

Fig. S4-3 ^{13}C NMR spectrum of **4α**

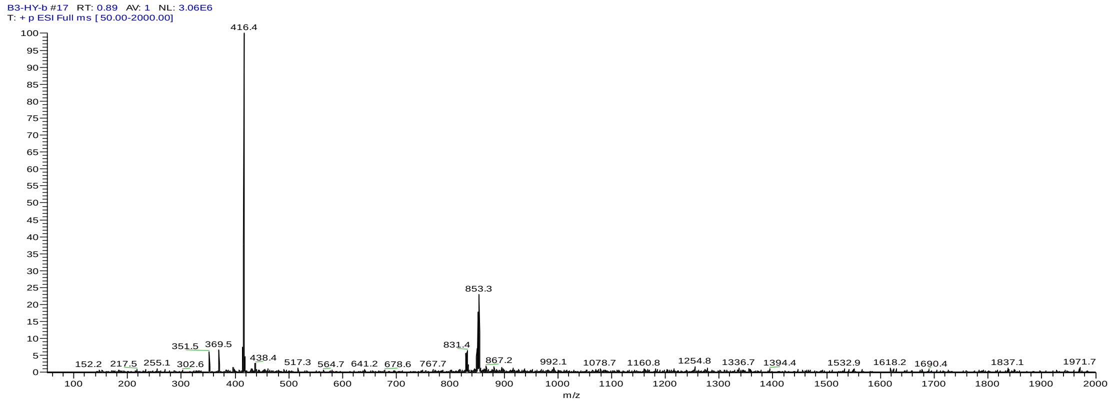

Fig. S5-1 ESI-MS of **4β**

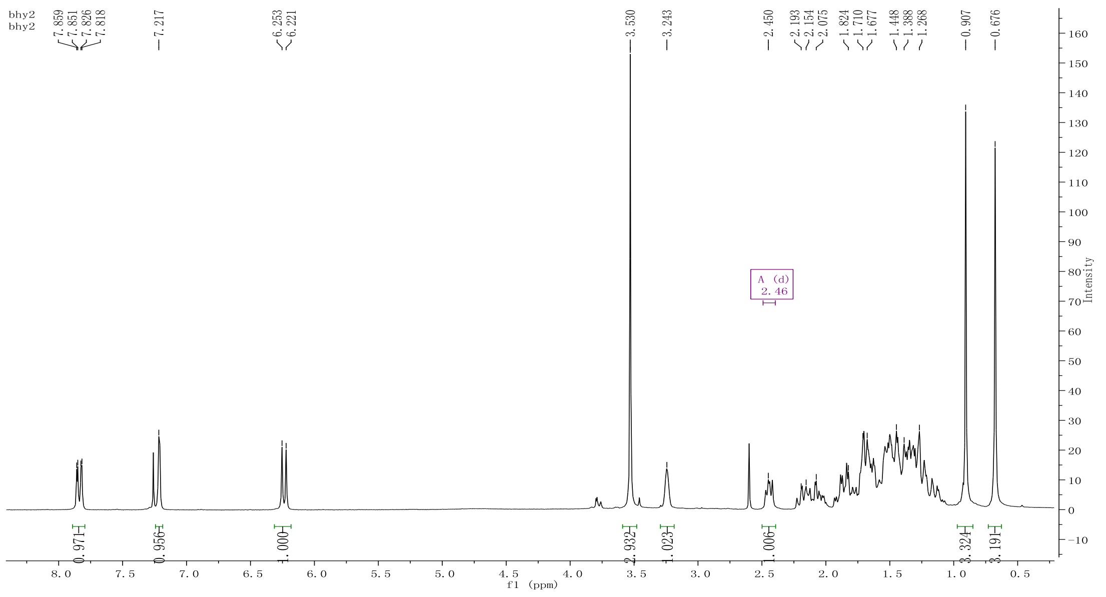

Fig. S5-2 ^{1}H NMR spectrum of **4β**

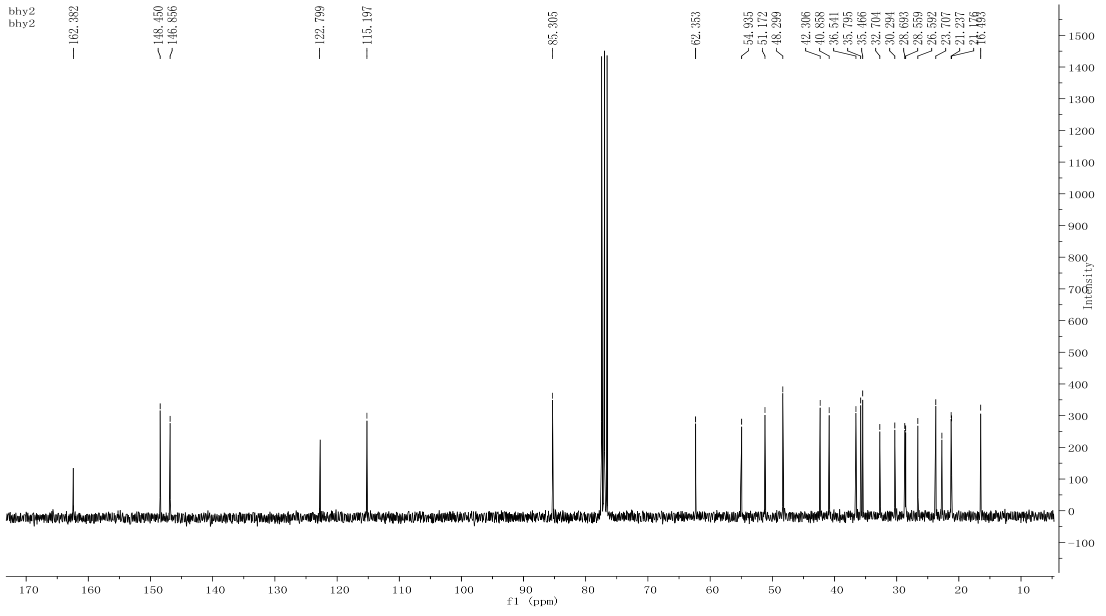

Fig. S5-3 ^{13}C NMR spectrum of **4β**

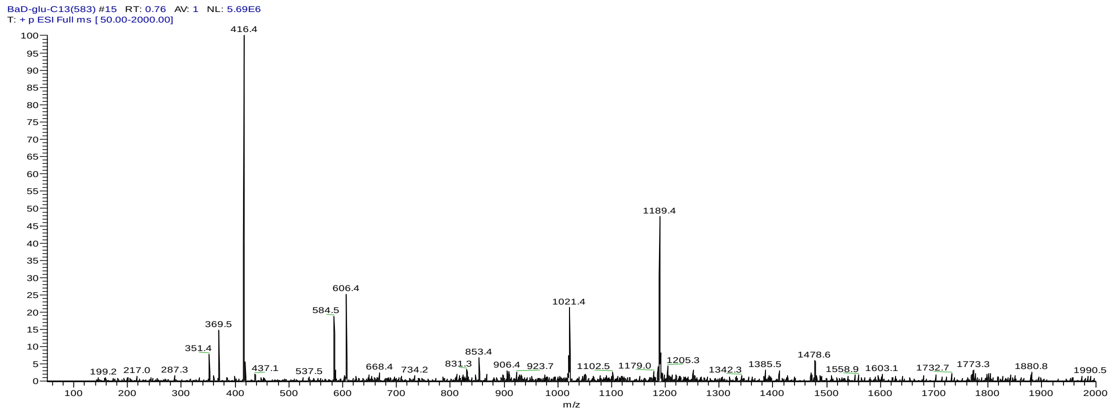

Fig. S6-1 ESI-MS of **5α**

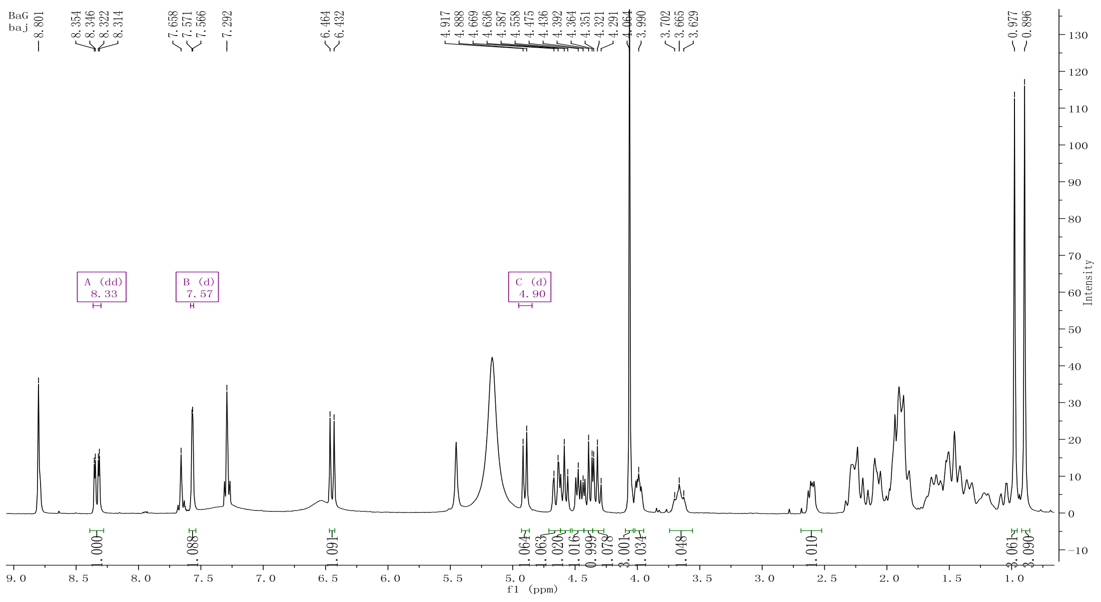

Fig. S6-2 ^{1}H NMR spectrum of **5α**

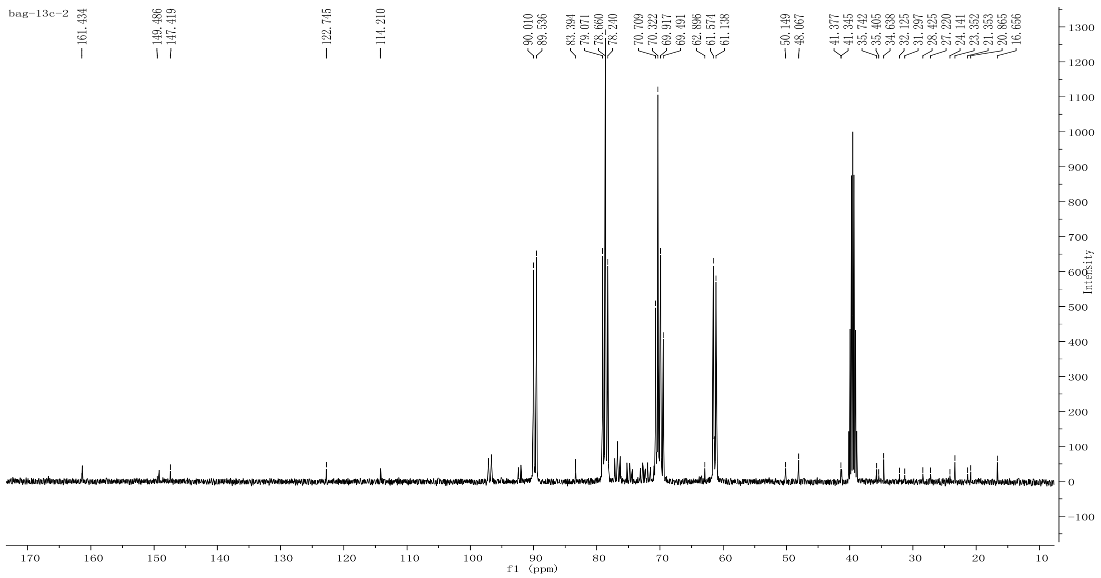

Fig. S6-3 ^{13}C NMR spectrum of **5α** (^{13}C labeled carbon is split by neighboring ^{13}C carbons into doublet or triplet)

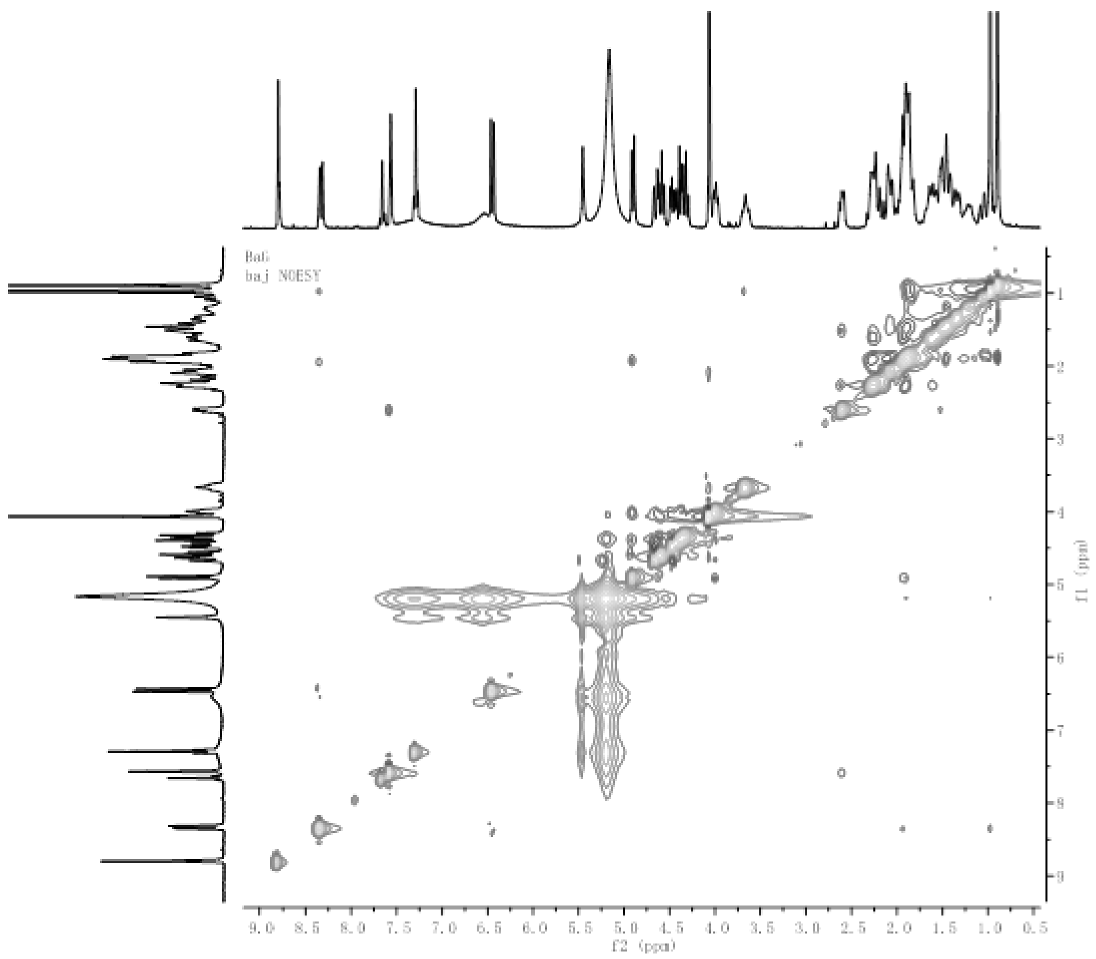

Fig. S6-4 NOESY spectrum of **5α**

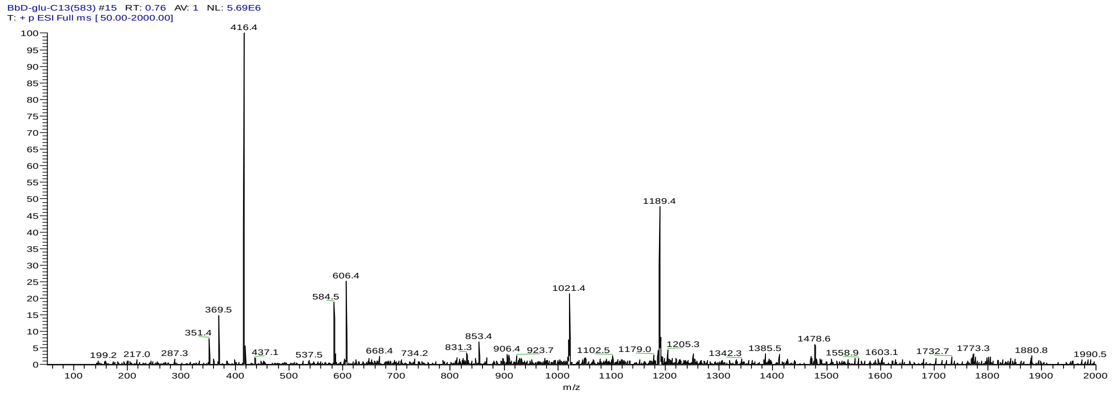

Fig. S7-1 ESI-MS of **5β**

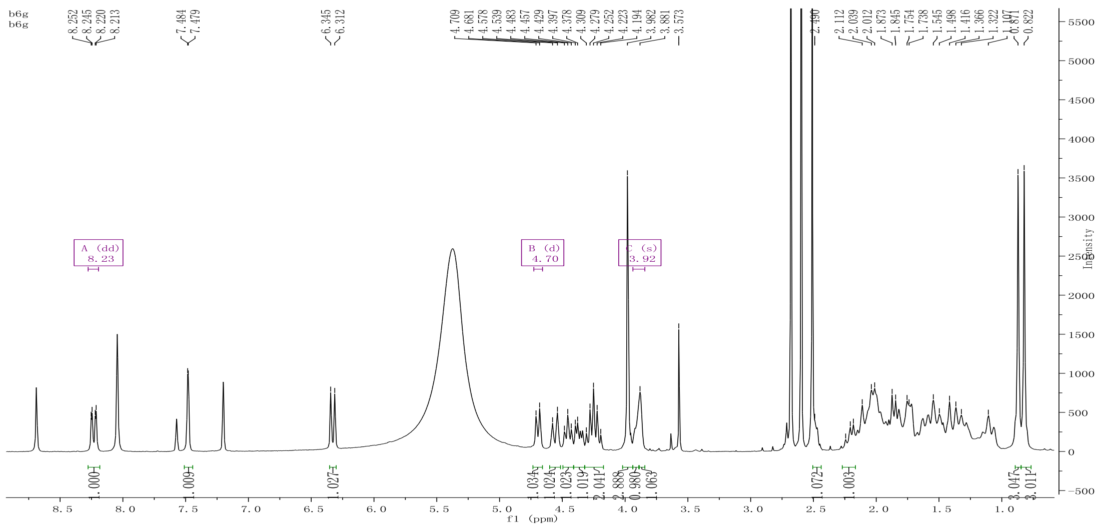

Fig. S7-2 ^{1}H NMR spectrum of **5β**

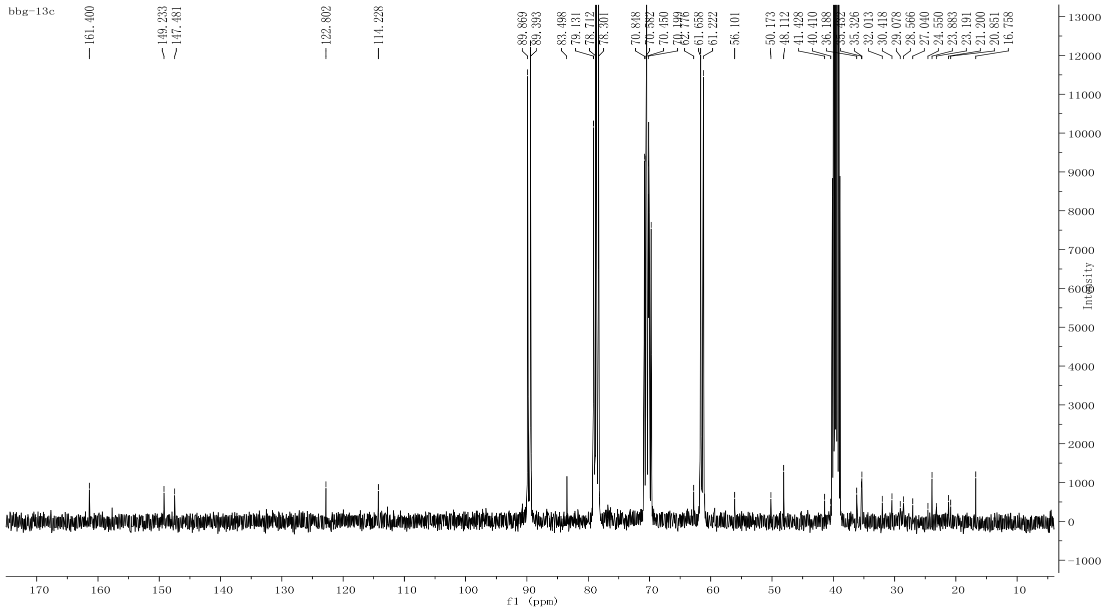

Fig. S7-3 ^{13}C NMR spectrum of **5β** (^{13}C labeled carbon is split by neighboring ^{13}C carbons into doublet or triplet)

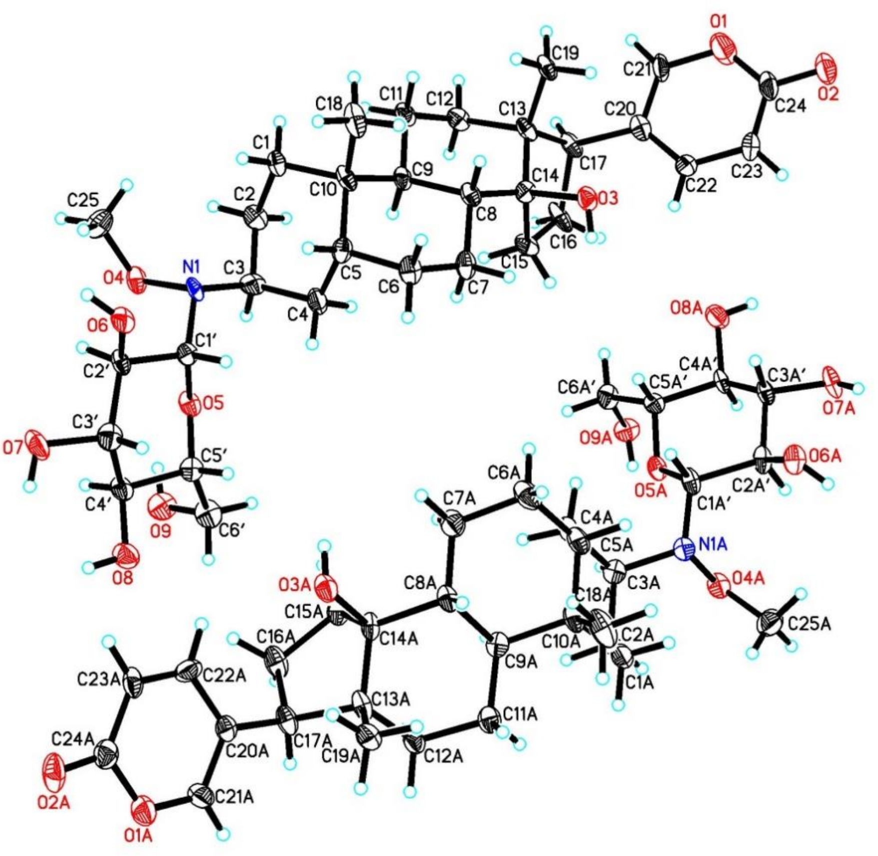

Fig. S7-4 X-ray structure of 5β showing the two molecules in the asymmetric unit

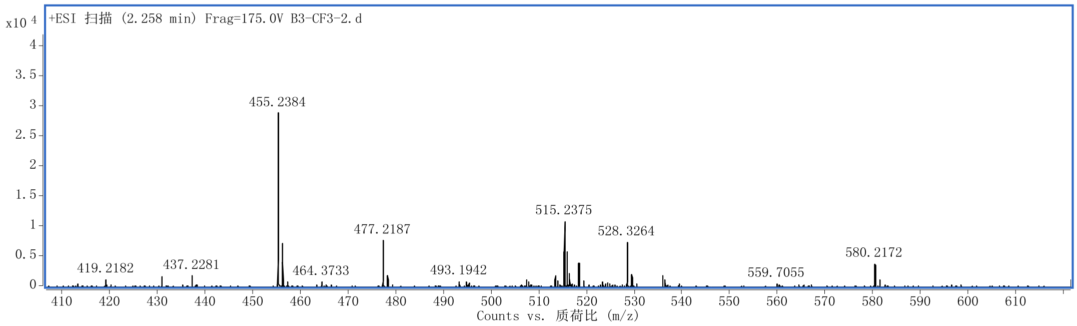

Fig. S8-1 HRESI-MS of **6α.**

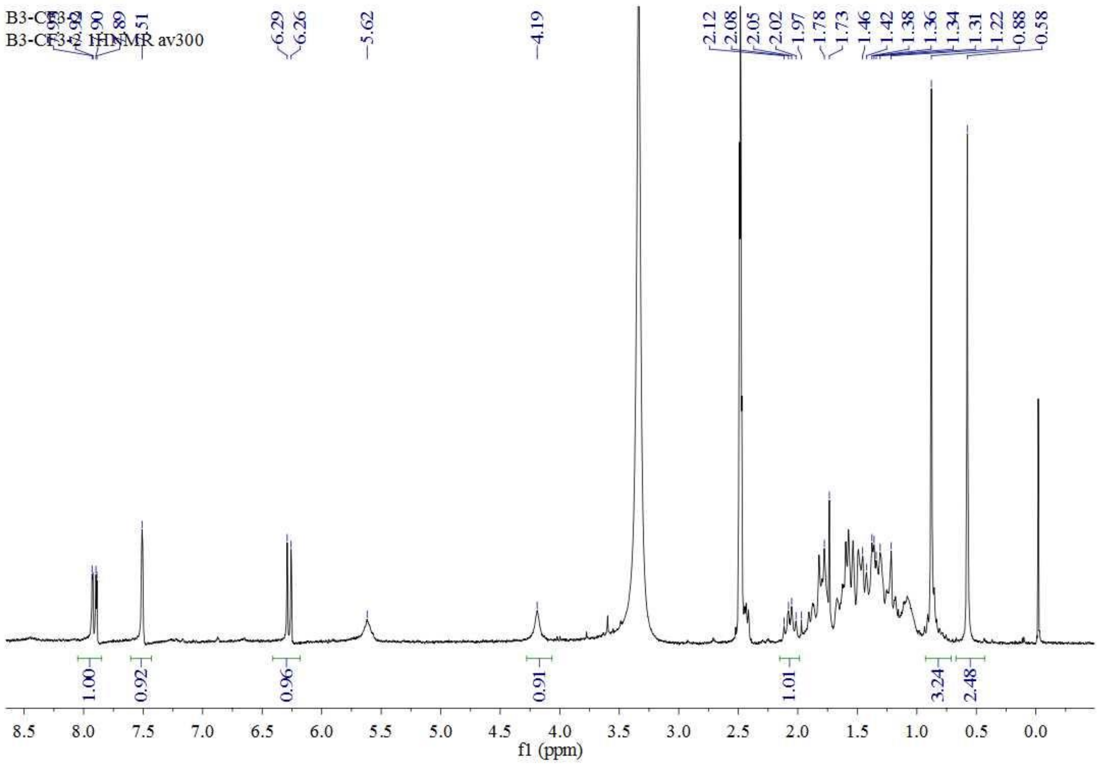

Fig. S8-2 ^{1}H-NMR of **6α.**

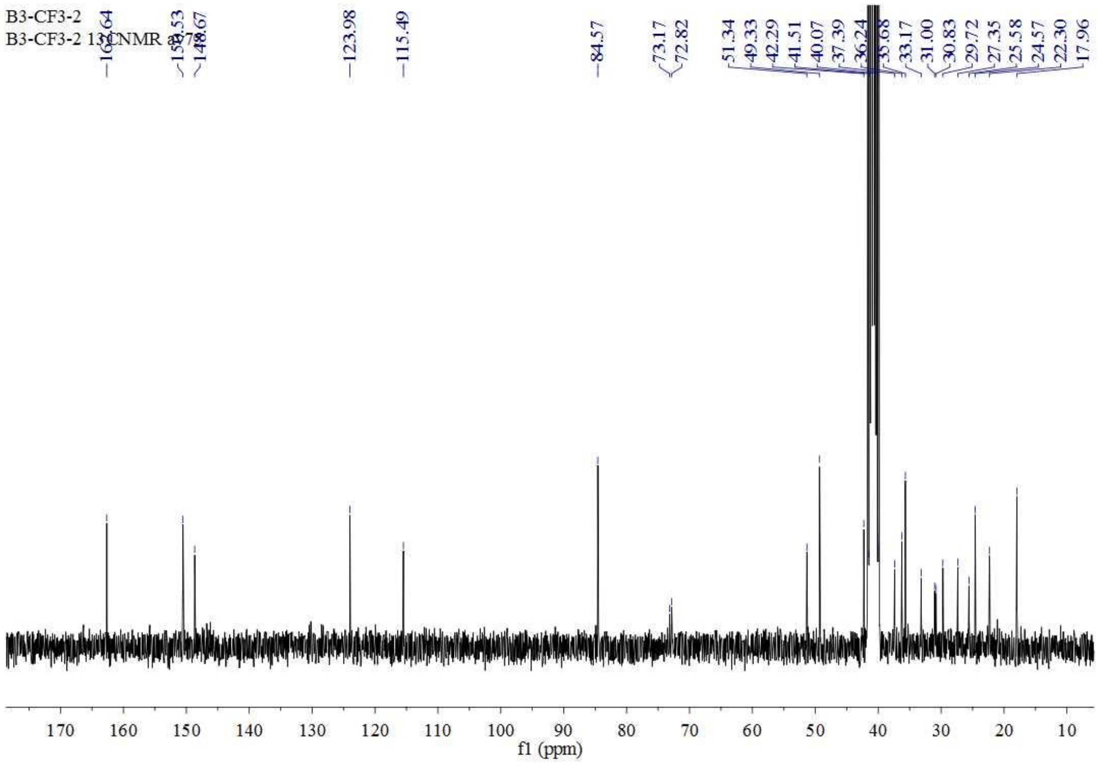

Fig. S8-3 ^{13}C-NMR of **6α.**

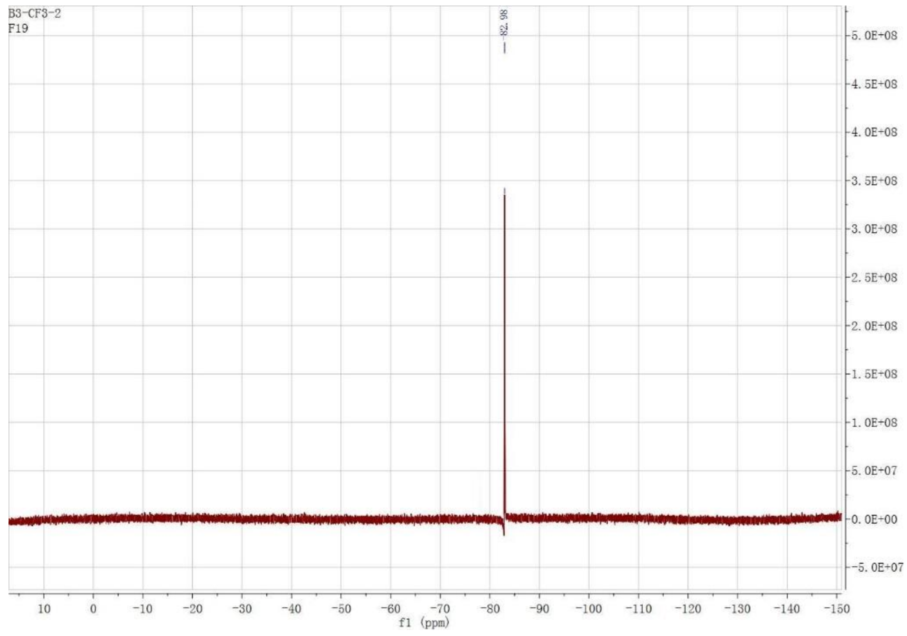

Fig. S8-4 ^{19}F NMR of **6α.**

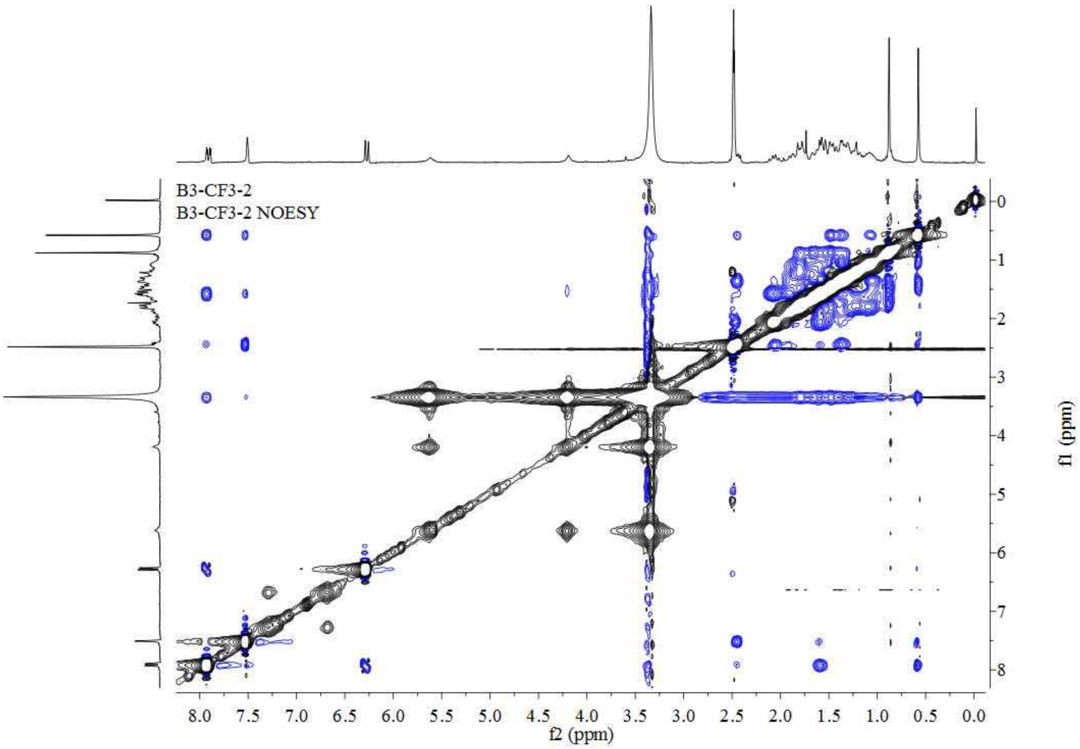

Fig. S8-5 NOESY spectrum of **6α.**

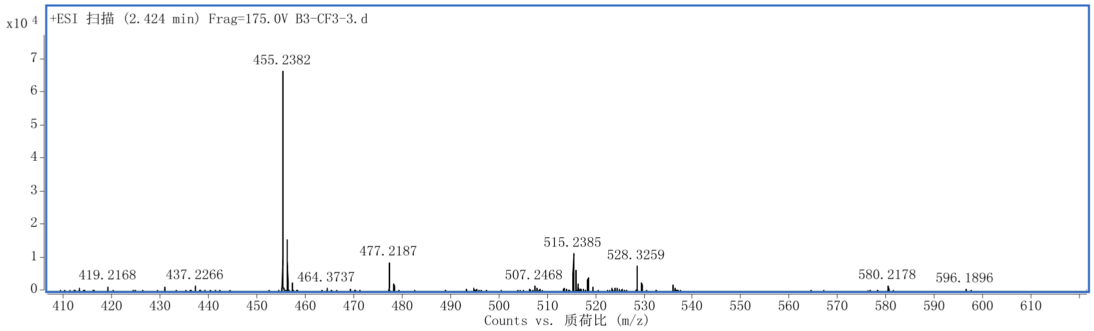

Fig. S9-1 HRESI-MS of **6β.**

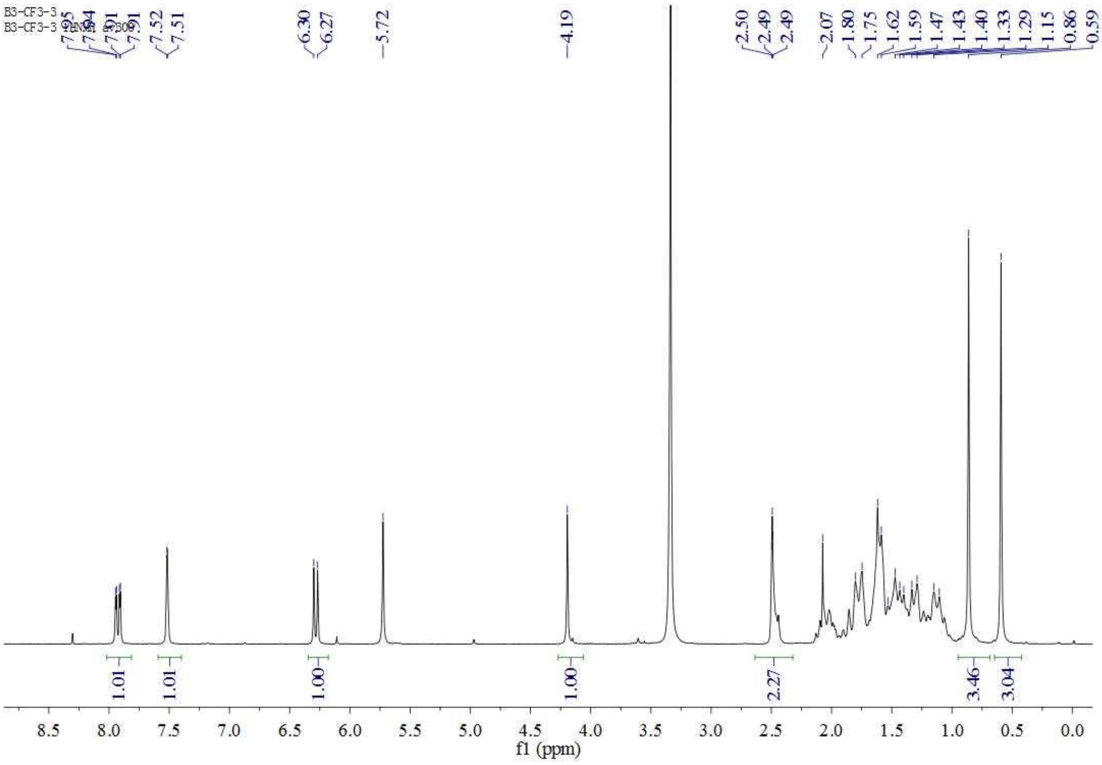

Fig. S9-2 ^{1}H-NMR of **6β.**

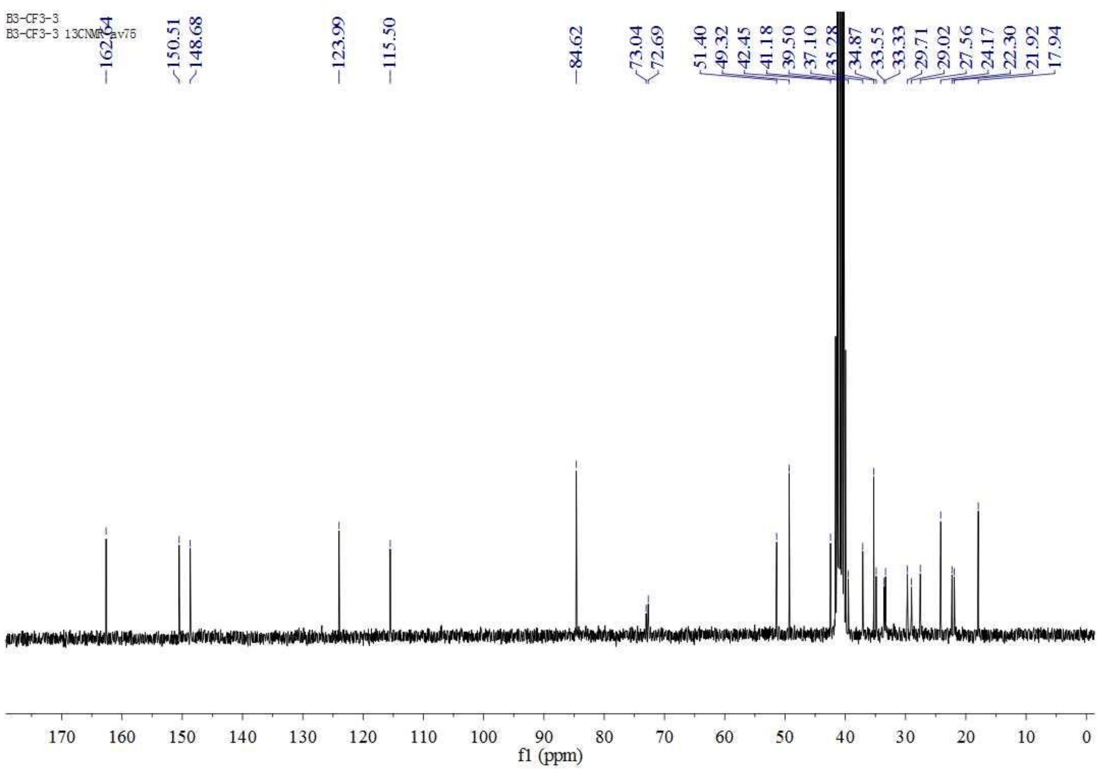

Fig. S9-3 ^{13}C-NMR of **6β.**

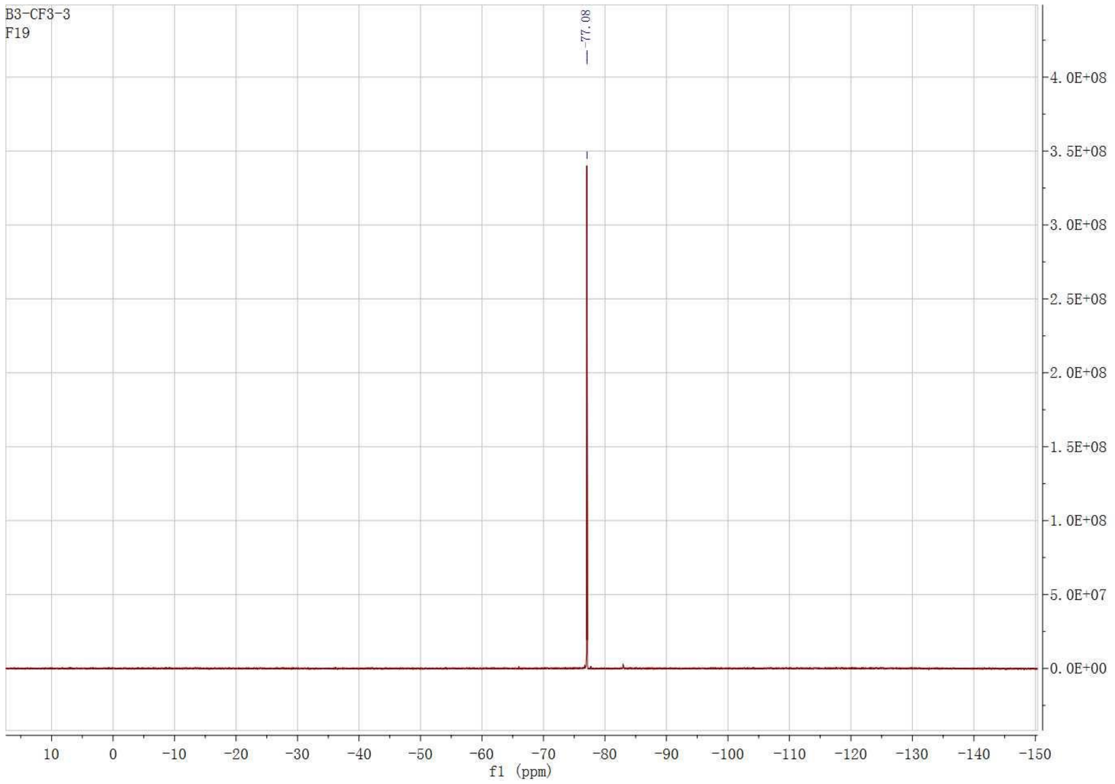

Fig. S9-4 ^{19}F NMR of **6β.**

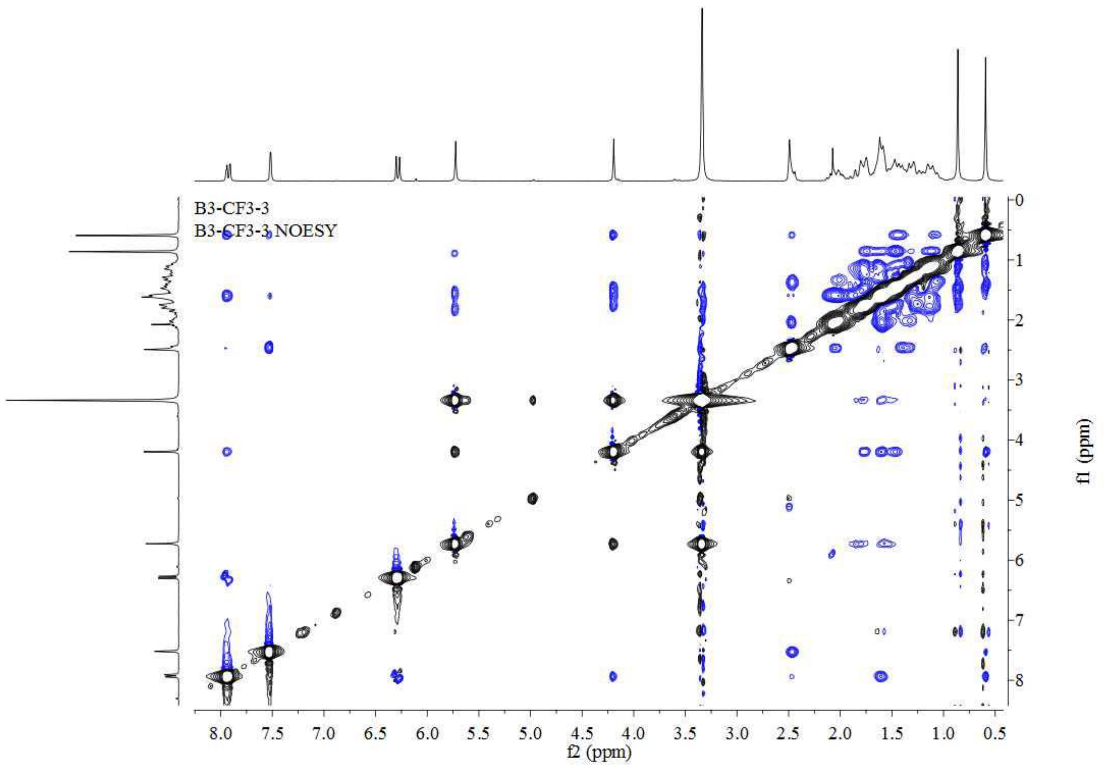

Fig. S9-5 NOESY Spectrum of **6β.**

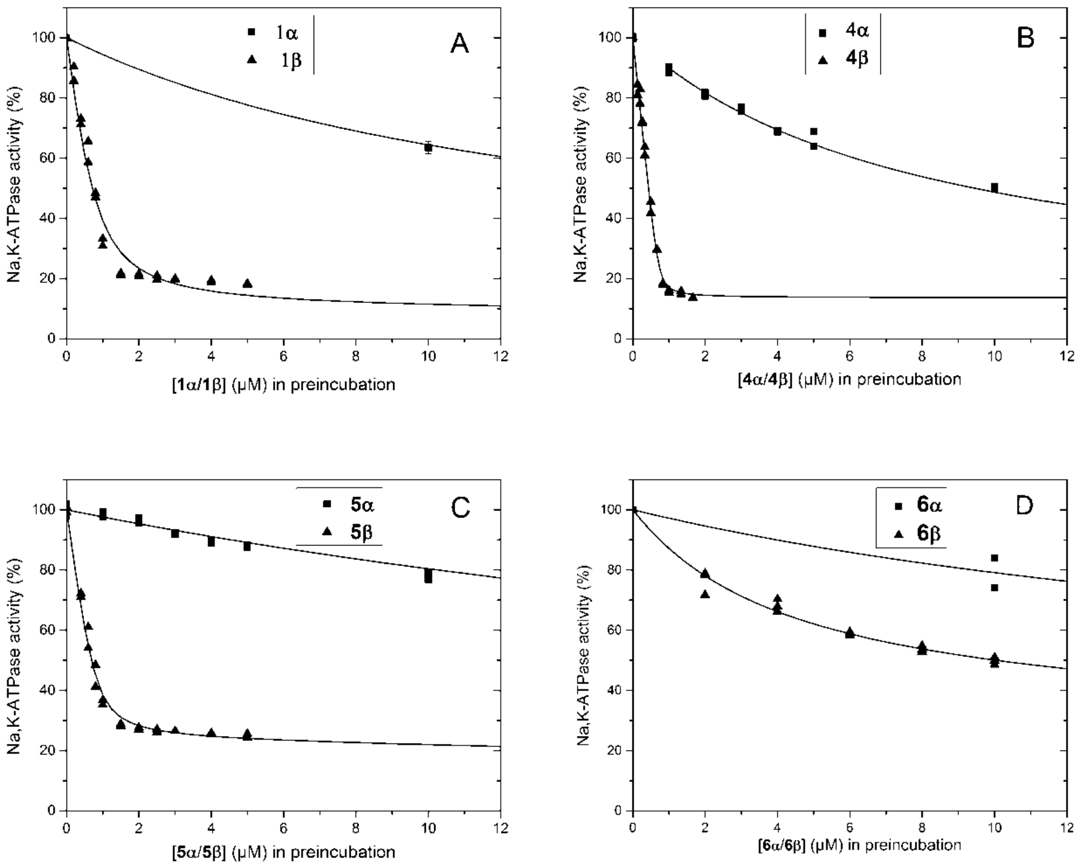

Figure S10. Expanded view of the dose-response curves in Figure 4 of the main text. All symbols and lines remain as described in the Figure 4 legend.

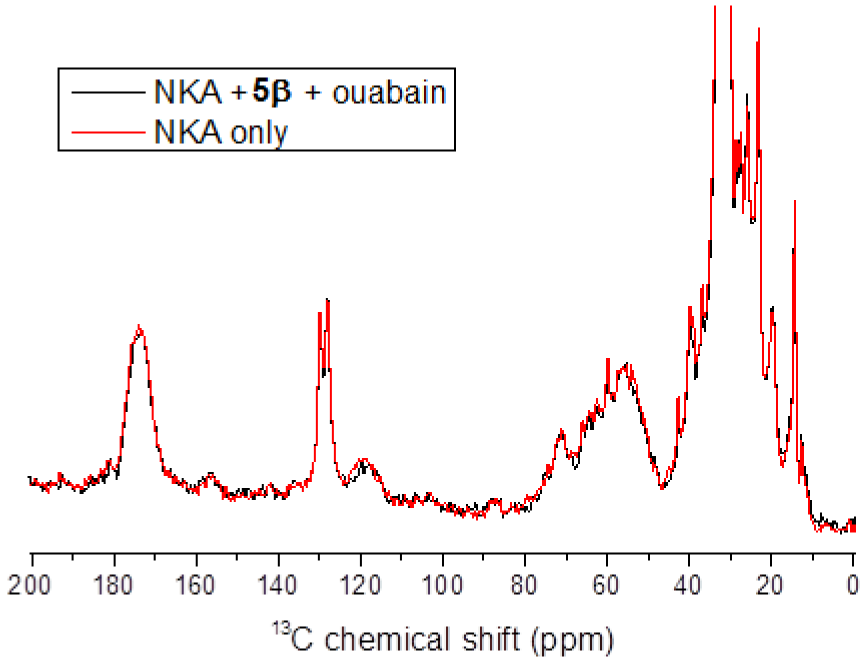

Fig S11-1. ^{13}C CPMAS SSNMR spectrum of NKA membrane preparations with **5β** in the presence of ouabain (red) and in the absence of **5β** (black).

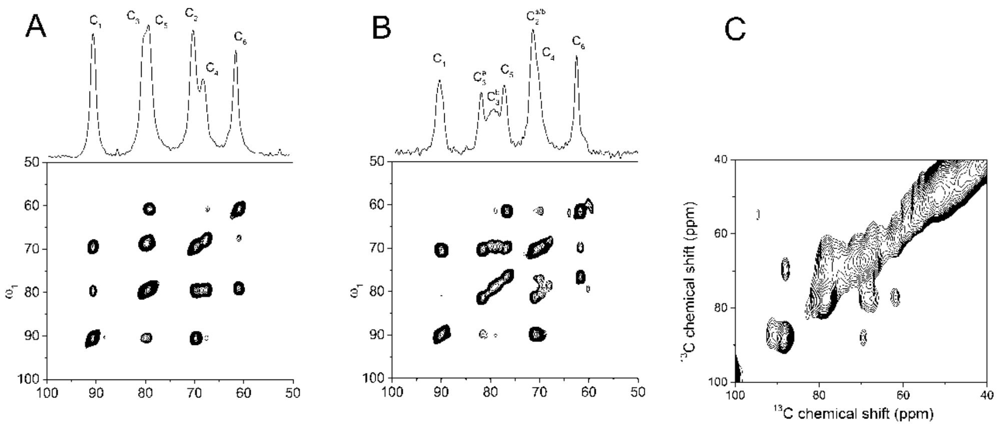

Fig S11-2. DARR solid-state NMR spectra of **5α** in the solid-state (A), **5β** in the solid-state (B) and the NKA-**5β** complex (C).

Detailed hydrogen bonds in compounds **1α**,**1β, 4α, 4β**, **5α** and **5β**

H-bonds of Compound **1α**:

Function group of 1	Residue	H-bond type	Distance (Å)
C-14 OH	T797	O---H-O	1.72

H-bonds of Compound **1β**:

Function group of 2	Residue	H-bond type	Distance (Å)
C-14 OH	T797	O---H-O	1.75
C-3 OH	E117	O-H---O	1.87

H-bonds of Compound **4α**:

Function group of 2	Residue	H-bond type	Distance (Å)
C-14 OH	T797	O---H-O	1.77
C-14 OH	T797	O-H---O	2.24
C-14 OH	D121	O-H---O	1.82

H-bonds of Compound **4β**:

Function group of 2	Residue	H-bond type	Distance (Å)
C-14 OH	T797	O---H-O	1.77
C-14 OH	T797	O-H---O	2.24
C-14 OH	D121	O-H---O	1.82
C-3 NH	D884	N-H---O	2.20

H-bonds of Compound **5α**:

Function group of 2	Residue	H-bond type	Distance (Å)
C-14 OH	T797	O---H-O	1.71
C-14 OH	T797	O-H---O	1.94
C-14 OH	D121	O-H---O	2.32
C-6’ OH	E117	O-H---O	1.89

H-bonds of Compound **5β**:

Function group of 2	Residue	H-bond type	Distance (Å)
C-14 OH	T797	O---H-O	2.26
C-14 OH	T797	O-H---O	2.11
C-14 OH	D121	O-H---O	2.04
C-2’ OH	Q111	O-H---O	1.78
C-2’ OH	E116	O-H---O	1.97
C-4’ OH	T114	O-H---O	2.13
C-6’ OH	E312	O-H---O	1.89
